# The Roles of the Two-Component System, MtrAB, in Response to Diverse Cell Envelope Stresses in *Dietzia* sp. DQ12-45-1b

**DOI:** 10.1128/aem.01337-22

**Published:** 2022-10-03

**Authors:** Xiaoyu Qin, Kaiduan Zhang, Yong Nie, Xiao-Lei Wu

**Affiliations:** a College of Engineering, Peking University, Beijing, China; b Institute of Ocean Research, Peking University, Beijing, China; c Institute of Ecology, Peking University, Beijing, China; Shanghai Jiao Tong University

**Keywords:** *Dietzia* sp. DQ12-45-1b, two-component system, MtrAB, stress, response, antibiotic resistance, cell morphology

## Abstract

Two-component systems (TCSs) act as common regulatory systems allowing bacteria to detect and respond to multiple environmental stimuli, including cell envelope stress. The MtrAB TCS of Actinobacteria is critical for cell wall homeostasis, cell proliferation, osmoprotection, and antibiotic resistance, and thus is found to be highly conserved across this phylum. However, how precisely the MtrAB TCS regulates cellular homeostasis in response to environmental stress remains unclear. Here, we show that the MtrAB TCS plays an important role in the tolerance to different types of cell envelope stresses, including environmental stresses (i.e., oxidative stress, lysozyme, SDS, osmotic pressure, and alkaline pH stresses) and envelope-targeting antibiotics (i.e., isoniazid, ethambutol, glycopeptide, and β-lactam antibiotics) in *Dietzia* sp. DQ12-45-1b. An *mtrAB* mutant strain exhibited slower growth compared to the wild-type strain and was characterized by abnormal cell shapes when exposed to various environmental stresses. Moreover, deletion of *mtrAB* resulted in decreased resistance to isoniazid, ethambutol, and β-lactam antibiotics. Further, Cleavage under targets and tagmentation sequencing (CUT&Tag-seq) and electrophoretic mobility shift assays (EMSAs) revealed that MtrA binds the promoters of genes involved in peptidoglycan biosynthesis (*ldtB*, *ldtA*, *murJ*), hydrolysis (GJR88_03483, GJR88_4713), and cell division (*ftsE*). Together, our findings demonstrated that the MtrAB TCS is essential for the survival of *Dietzia* sp. DQ12-45-1b under various cell envelope stresses, primarily by controlling multiple downstream cellular pathways. Our work suggests that TCSs act as global sensors and regulators in maintaining cellular homeostasis, such as during episodes of various environmental stresses. The present study should shed light on the understanding of mechanisms for bacterial adaptivity to extreme environments.

**IMPORTANCE** The multilayered cell envelope is the first line of bacterial defense against various extreme environments. Bacteria utilize a large number of sensing and regulatory systems to maintain cell envelope homeostasis under multiple stress conditions. The two-component system (TCS) is the main sensing and responding apparatus for environmental adaptation. The MtrAB TCS highly conserved in Actinobacteria is critical for cell wall homeostasis, cell proliferation, osmoprotection, and antibiotic resistance. However, how MtrAB works with regard to signals impacting changes to the cell envelope is not fully understood. Here, we found that in the Actinobacterium *Dietzia* sp. DQ12-45-1b, a TCS named MtrAB is pivotal for ensuring normal cell growth as well as maintaining proper cell morphology in response to various cell envelope stresses, namely, by regulating the expression of cell envelope-related genes. Our findings should greatly advance our understanding of the adaptive mechanisms responsible for maintaining cell integrity in times of sustained environmental shocks.

## INTRODUCTION

Microorganisms inhabit diverse environments, often enduring conditions too extreme for other life forms to exist. The adaptive responses of microorganisms to environmental conditions lay the foundations for microbial life and its function in the global geobiochemical cycle, its use in bioremediation, and bioindustry, as well as its role in pathogenesis.

The cell envelope constitutes the first line of defense against life-threatening external stress. The roles of the cell envelope include keeping the integrity of the cell, selectively transporting nutrients and waste products, maintaining cellular homeostasis, and protecting the cell from harmful environmental conditions. The envelope of Gram-negative bacteria is composed of an outer membrane, a periplasm containing a peptidoglycan layer, and an inner membrane. In contrast, the cell envelope of Gram-positive bacteria consists of 2 functional layers; a cytoplasmic membrane, and a cell wall, except for the order Corynebacteriales. Compared with other Gram-positive bacteria, species from the order Corynebacteriales, which includes the important pathogen Mycobacterium, the production strain *Corynebacterium*, and the environmental strain *Dietzia*, are characterized by a more complex multi-layered cell envelope. These species possess an additional polysaccharide layer consisting of arabinogalactan and an outer membrane layer composed of mycolic acids (1, 2).

Certain stresses can disturb the integrity of the cell envelope, and therefore threaten the survival of the cell. Such stresses are referred to as cell envelope stresses and they include environmental types of stresses (e.g., temperature shocks, changing pH, osmotic stress, oxidative stress, and envelope-targeting antibiotics) as well as cell-intrinsic types of stresses (e.g., protein misfolding, mutation, secretion defects, and membrane disruption) (3, 4). Thus, the survival of microorganisms critically depends on their ability to sense envelope stresses, activate stress responses, and ultimately restore envelope homeostasis.

To respond to cell envelope stresses in a timely and proportionate manner, microorganisms have developed various defense systems. One of the best-known regulatory systems responsible for cell envelope stress sensing and response are so-called two-component systems (TCSs) (3). Prototypical TCSs typically consist of a histidine kinase (HK) serving as a stress sensor and a response regulator (RR) serving as a regulatory effector. The cell envelope stress-responsive TCSs can be activated by cell envelope stress signals, such as the integrity of the cell envelope (3, 5, 6). These activated TCSs commonly serve as global regulators to maintain cellular homeostasis. One example of such TCSs is the CpxAR identified earlier in Escherichia coli. The CpxAR TCS consists of a histidine kinase CpxA and a regulatory protein CpxR. This system is activated by signals that negatively affect the functioning of the cell envelope such as alkaline pH, overexpression of specific envelope protein, high osmolarity, and perturbations in membrane structure (4, 7, 8). The regulon of the CpxAR TCS consists of more than 100 different genes, many of which encode factors in protein folding and degradation, and proteins involved in efflux, biofilm formation, and antibiotic resistance (9, 10). Another example of cell envelope stress-responsive TCSs is the non-orthodox Rcs TCS found in Enterobacteriaceae. This TCS was shown to be activated via the outer membrane or peptidoglycan perturbation, lipopolysaccharide (LPS) synthesis defects, lipoprotein mislocalization, osmotic and oxidative stress, and β-lactam antibiotics. The Rcs TCS subsequently regulates downstream genes involved in virulence, capsule biosynthesis, biofilm formation, and motility (11–13).

While a considerable number of cell envelope stress-responsive TCSs have been described, few studies have so far focused on TCSs that regulate cell envelope homeostasis directly in response to stresses targeting the cell envelope. For example, the WalKR TCS, which is highly conserved in low G+C Gram-positive bacteria, was found to be induced by antibiotics targeting the late stages of peptidoglycan synthesis. The WalKR regulon includes a large number of genes that participate in cell wall biosynthesis, metabolism, and cell division (14, 15). In Vibrio cholerae, the WigKR TCS controls cell envelope homeostasis in response to cell wall stress induced by various antibiotics (16). Notably, similar cell envelope stress response systems have not been reported in Corynebacteriales, which are characterized by the cell envelope of complex composition.

The MtrAB TCS, which is conserved in Actinobacteria, plays multiple roles in cell wall metabolism, cell division, DNA replication, and cell proliferation (17–19). In Mycobacterium tuberculosis, MtrA expression is downregulated when cells encounter envelope stresses such as SDS and DETA-NO (17). Importantly, M. tuberculosis requires MtrB to withstand acid stress (pH 5.5) and hypoxia (20). Moreover, the MtrAB TCS plays a critical role in the osmoregulation and susceptibility to cell wall-targeting antibiotics in Corynebacterium glutamicum (21). Together, these findings suggested that the MtrAB TCS is important for the response to specific envelope stresses. However, which cell envelope stress responses are related to MtrAB has remained unclear. Previous studies using M. tuberculosis reported that its MtrA directly regulates cell envelope homeostasis via peptidoglycan synthetase genes *ftsI*, *dacB1*, *wag31*, peptidoglycan hydrolase genes *ripA*, *rpfA-E*, and genes involved in mycolic acids assembly (*fbpB*, *fbpC*) (17). MtrB can interact with peptidoglycan synthetase FtsI, Wag31, and cell envelope biosynthesis regulators PknA and PknB (22). In comparison, the peptidoglycan hydrolase genes *mepA*, *nlpC*, and *rpf2* are directly repressed by MtrA in C. glutamicum (23). In addition, deletion of either MtrA or MtrB in both Mycobacteria and Corynebacteria alters their cell morphology, suggesting that the MtrAB TCS affects cell envelope homeostasis (17, 21, 24). Therefore, cell envelope stress response of MtrAB and cell envelope homeostasis in Actinobacteria are possibly linked at a mechanistic level.

The actinobacterial genus *Dietzia* is widely distributed in diverse habitats, such as soda lakes (25, 26), deep seas (27, 28), the soil of deserts (29, 30), oil fields (31, 32), the surface of plants (33), and clinical samples (34–36). Several *Dietzia* strains are potential human pathogens in immunocompetent and immunocompromised patients (37–39). As most *Dietzia* species can degrade a wide range of alkanes and aromatic compounds, and they can tolerate high alkaline and salty environments (40), they have great potential for bioremediation in alkaline and salty environments (41). Moreover, *Dietzia* species have many applications in the food, medical, and chemical industries (40, 42). Interestingly, some *Dietzia* species are thought to be potential probiotics to reduce Mycobacterium avium *paratuberculosis* (MAP) (43, 44).

Here, we set out to investigate the effects of deleting *mtrAB* genes on the growth and cell morphology under the cell envelope, ultimately aiming to better understand the role of the MtrAB TCS in cell envelope stress resistance in *Dietzia* sp. DQ12-45-1b. Using CUT&Tag-seq combined with electrophoretic mobility shift assays (EMSAs), we constructed a regulatory network of MtrAB in *Dietzia* sp. DQ12-45-1b. In summary, our findings reveal that the MtrAB TCS plays a critical role in the response to a variety of cell envelope stresses and in maintaining cell envelope homeostasis.

## RESULTS

### Deletion of the *mtrAB* genes decreases the growth of *Dietzia* sp. DQ12-45-1b under diverse envelope stresses.

**FIG 1:**
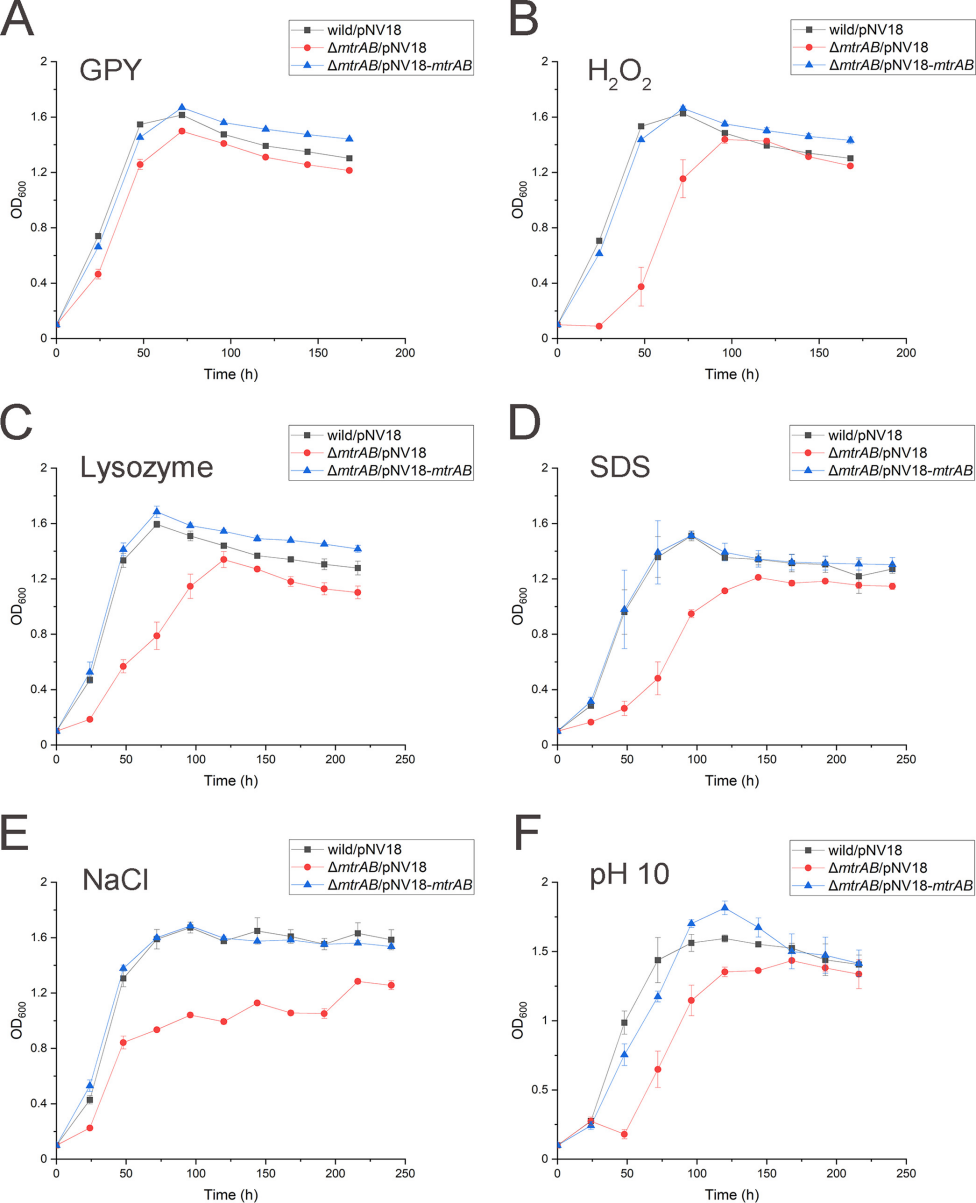
Growth curves of *Dietzia* sp. DQ12-45-1b wild-type strain carrying pNV18, Δ*mtrAB* carrying pNV18 (empty plasmid), pNV18-*mtrAB* under different stresses. (A) GPY medium; (B) GPY medium with 0.0025% H_2_O_2_; (C) GPY medium with 2 μg/mL lysozyme; (D) GPY medium with 0.0005% SDS; (E) GPY medium with 0.625M NaCl; (F) pH 10 GPY medium. The results shown represent the mean values (±SD) of three replicates.

Based on the phylogenetic analysis of MtrAB in *Corynebacterineae* and *Streptomycineae* suborders, we found that both MtrA and MtrB phylogenetic trees consisted of 4 distinct clusters. Sequences from the families *Dietziaceae* and *Corynebacteriaceae* of the suborder *Corynebacterineae* formed two clusters that were distinct from the sequences of other *Corynebacterineae* species (Fig. S1 and S2). The results suggested that the MtrAB TCS in the family of *Dietziaceae* has unique functions compared to other families of *Corynebacterineae* suborder. To investigate whether the MtrAB TCS of *Dietzia* sp. DQ12-45-1b is involved in bacterial adaption to cell envelope stresses, we compared the growth of *Dietzia* sp. DQ12-45-1b and its *mtrAB* mutant strain under a number of conditions causing envelope stress. As shown in Fig. 1, the inactivation of *mtrAB* genes significantly suppressed the growth of *Dietzia* sp. DQ12-45-1b under a range of stresses, including oxidative stress, lysozyme, SDS, osmotic pressure, and alkaline stress. The *mtrAB* complementary strains exhibited similar growth to that of the wild-type strain under all culture conditions (Fig. 1). Together, these findings suggested that MtrAB TCS plays an important role in cellular resistance to diverse cell envelope-related stress conditions. Moreover, we found that all strains (i.e., the wild-type, Δ*mtrAB* mutant, and *mtrAB* complementary strains) exhibited near-identical growth patterns when grown in glucose peptone yeast (GPY) medium (Fig. 1A), which is an optimal medium for *Dietzia* spp. (45). These results suggested that MtrAB remains inactive in the absence of environmental stress.

### Deletion of *mtrAB* affects the susceptibility of *Dietzia* sp. DQ12-45-1b to antibiotics.

To investigate the effects of deletion of MtrAB TCS in antibiotic sensitivity in *Dietzia* sp. DQ12-45-1b, we determined the minimum inhibitory concentrations (MICs) of envelope-targeting antibiotics including isoniazid, ethambutol, glycopeptide (i.e., vancomycin), and β-lactam antibiotics (i.e., ampicillin, cephalexin, and penicillin) against *Dietzia* sp. DQ12-45-1b wild-type, the Δ*mtrAB* mutant, as well as the complementary strains. The Δ*mtrAB* mutant strain showed a 2-fold decrease in the MICs of ethambutol, a 4-fold decrease in the MICs of cephalexin and penicillin, and an 8-fold decrease in the MICs of isoniazid and ampicillin compared to the wild-type strain. In comparison, the Δ*mtrAB* mutant strain showed a 2-fold increase in the MICs of vancomycin compared with the wild-type strain (Table 1). Together, these results suggested that MtrAB TCS contributes to establishing resistance against cell envelope-targeting antibiotics, especially antibiotics targeting mycolic acids (isoniazid) and penicillin-binding proteins (β-lactam antibiotics).

**TABLE 1:** MICs for different antibiotics of *Dietzia* sp. DQ12-45-1b wild/pNV18, Δ*mtrAB*/pNV18, and Δ*mtrAB*/pNV18-*mtrAB*

Antibiotics	MIC for *Dietzia* sp. DQ12-45-1b strains (μg/mL)			Fold change (WT/Δ)
	Wild/pNV18	Δ*mtrAB*/pNV18	Δ*mtrAB*/pNV18-*mtrAB*	
Isoniazid	4096	512	4096	8
Ethambutol	8	4	2	2
Vancomycin	0.125	0.25	0.25	0.5
Ampicillin	4	0.5	2	8
Cephalexin	8	2	4	4
Penicillin	2	0.5	2	4

### MtrAB TCS regulates peptidoglycan biosynthesis and cell division under envelope stress conditions.

**FIG 2:**
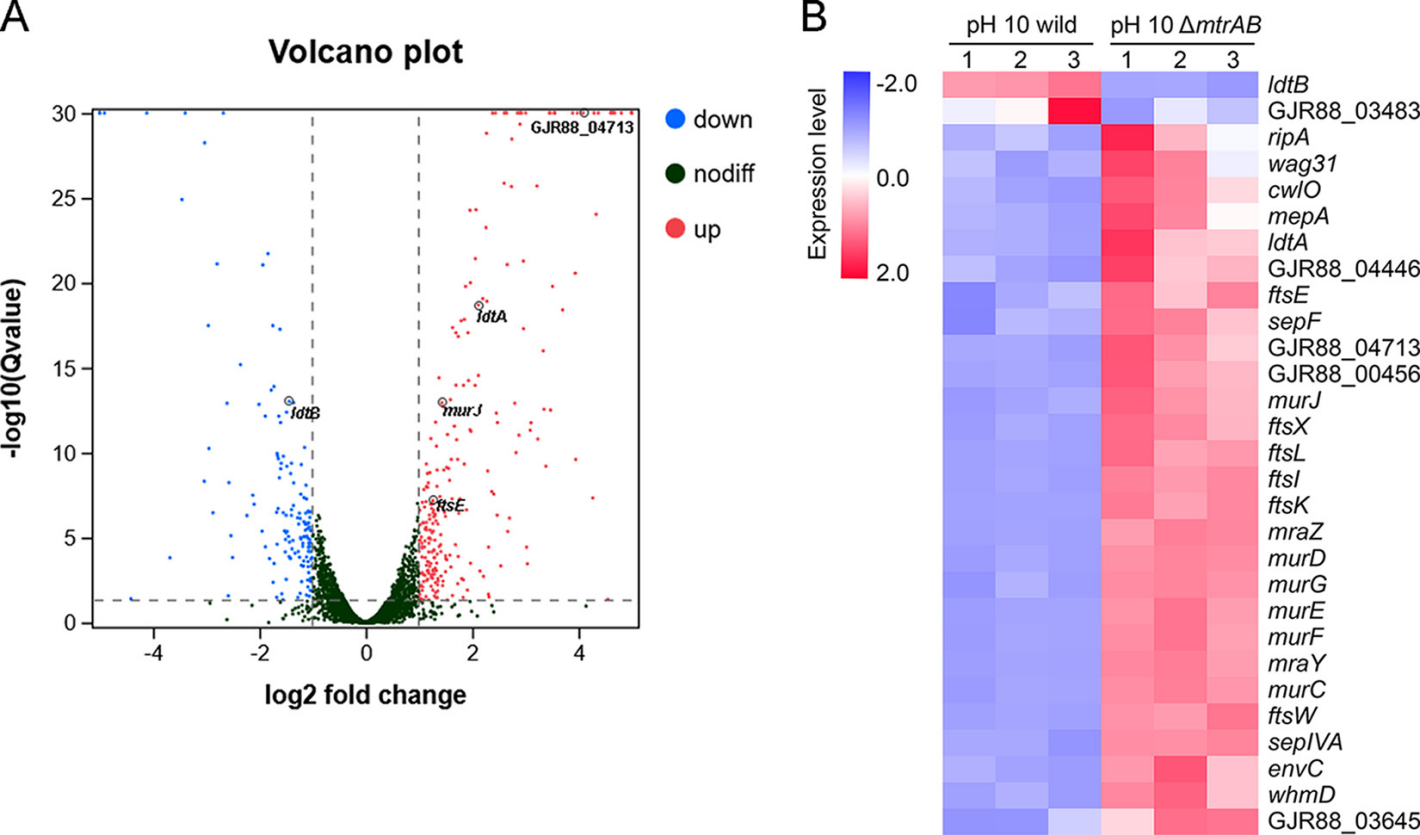
Comparison of transcriptional differences of *Dietzia* sp. DQ12-45-1b wild-type and Δ*mtrAB* mutant strains under the alkaline pH condition. (A) Volcano plot of the differentially expressed genes (DEGs). (B) Heatmap of the expression levels in DEGs involved in cell envelope homeostasis (fold change ≥ 2, FDR ≤ 0.05). The expression data were normalized by row Z-scores using FPKM values. Red represents upregulated genes, blue represents downregulated genes.

Together, these findings indicated that MtrAB TCS senses the extracellular stresses and maintains cellular homeostasis. To investigate how the inactivation of MtrAB affected the cellular response to cell envelope stresses, we compared transcript abundance in the wild-type and Δ*mtrAB* mutant strains under alkaline conditions, a typical representative of cell envelope stresses (4). As shown in Fig. 2A, 144 genes were upregulated and 253 genes were downregulated in the Δ*mtrAB* mutant when compared to the wild-type strain under alkaline stress conditions by the global analysis of differentially expressed genes (DEGs). Our KEGG enrichment analysis showed that 7 genes of the “peptidoglycan biosynthesis” pathway were induced in the Δ*mtrAB* mutant under alkaline stress (Fig. S3 and Table S1). We also found that the expression of a large number of genes involved in peptidoglycan biosynthesis (*murCDEFGJ*, *ftsW*, *ftsI*, *mraY*, *ldtA*, *ldtB*), hydrolysis (*cwlO*, *mepA*, *ripA*, GJR88_00456, GJR88_04713), cell division and elongation (*ftsEXLK*, *sepF*, *sepIVA*, *wag31*), and related regulators (*envC*, *whmD*, *mraZ*, GJR88_04446, GJR88_03645) significantly changed in the Δ*mtrAB* mutant compared with the wild-type strain when exposed to alkaline stress. However, at pH 8, these genes exhibited similar transcript levels in both the wild-type and Δ*mtrAB* mutant strains (Fig. 2B, Fig. S4, and Table S1). Together, these results were consistent with our findings, showing that the MtrAB TCS regulates cell envelope homeostasis during cell envelope stress conditions.

### Inactivation of *mtrAB* alters cell morphology under cell envelope stresses.

**FIG 3:**
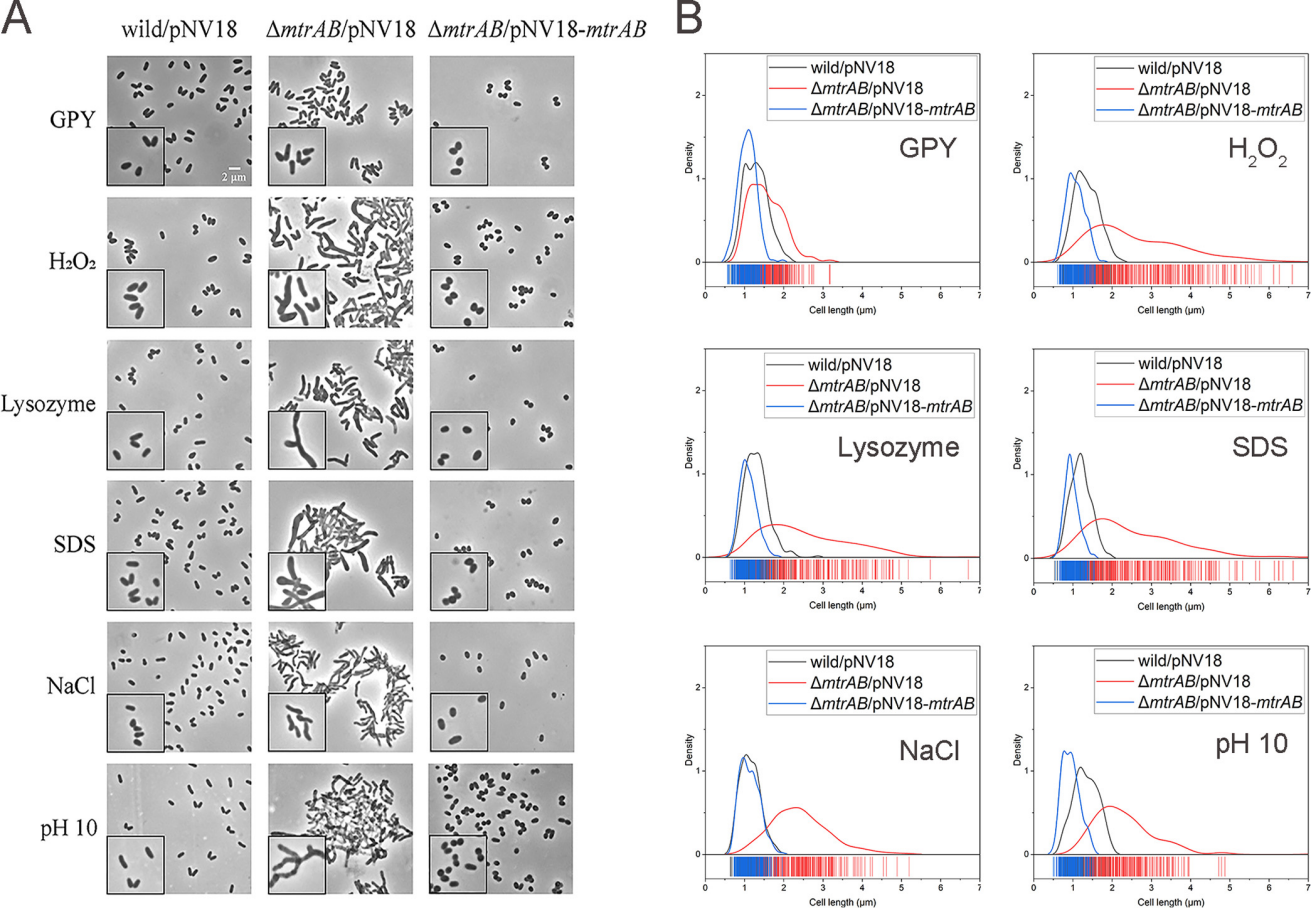
Cell morphology of *Dietzia* sp. DQ12-45-1b wild-type strain carrying pNV18, Δ*mtrAB* carrying pNV18 (empty plasmid), pNV18-*mtrAB* under different stresses. (A) The representative micrographs of the wild/pNV18, Δ*mtrAB*/pNV18, and Δ*mtrAB*/pNV18-*mtrAB* under various conditions. Inserts show a small portion of each image. (B) Distribution curves of the cell lengths of ~200 cells/sample from (A) were measured using the Digimizer software.

As the results in the previous section indicated that MtrAB regulates genes involved in peptidoglycan homeostasis and cell division, we next analyzed the cell shapes and measured the lengths of the wild-type, Δ*mtrAB* mutant, and complementary strains in response to different cell envelope stresses. Similarly, the wild-type, Δ*mtrAB* mutant, and complementary strains were cultured in oxidative, lysozyme, SDS, osmotic pressure, and alkaline pH stresses. When grown in GPY medium to the middle exponential phase, both the wild-type strain and the Δ*mtrAB* mutant exhibited short-rod shapes. We found that in response to oxidative, lysozyme, SDS, osmotic pressure, and alkaline pH stresses, the lengths of cells increased in the Δ*mtrAB* mutant strain, with the mean length of 1.15–1.59 μm for the wild-type strain and 2.31–2.75 μm for the Δ*mtrAB* mutant (Fig. 3A and B). In addition to increased lengths, the Δ*mtrAB* mutant cells showed irregular and branched shapes in response to cell envelope stresses (Fig. 3A). The complementary strain can restore these phenotypes of cell morphology. Together, these results confirmed that the MtrAB TCS regulates peptidoglycan metabolism and cell division to maintain cellular homeostasis in response to cell envelope stress conditions.

### Identification of candidate genes regulated by the regulator MtrA.

**FIG 4:**
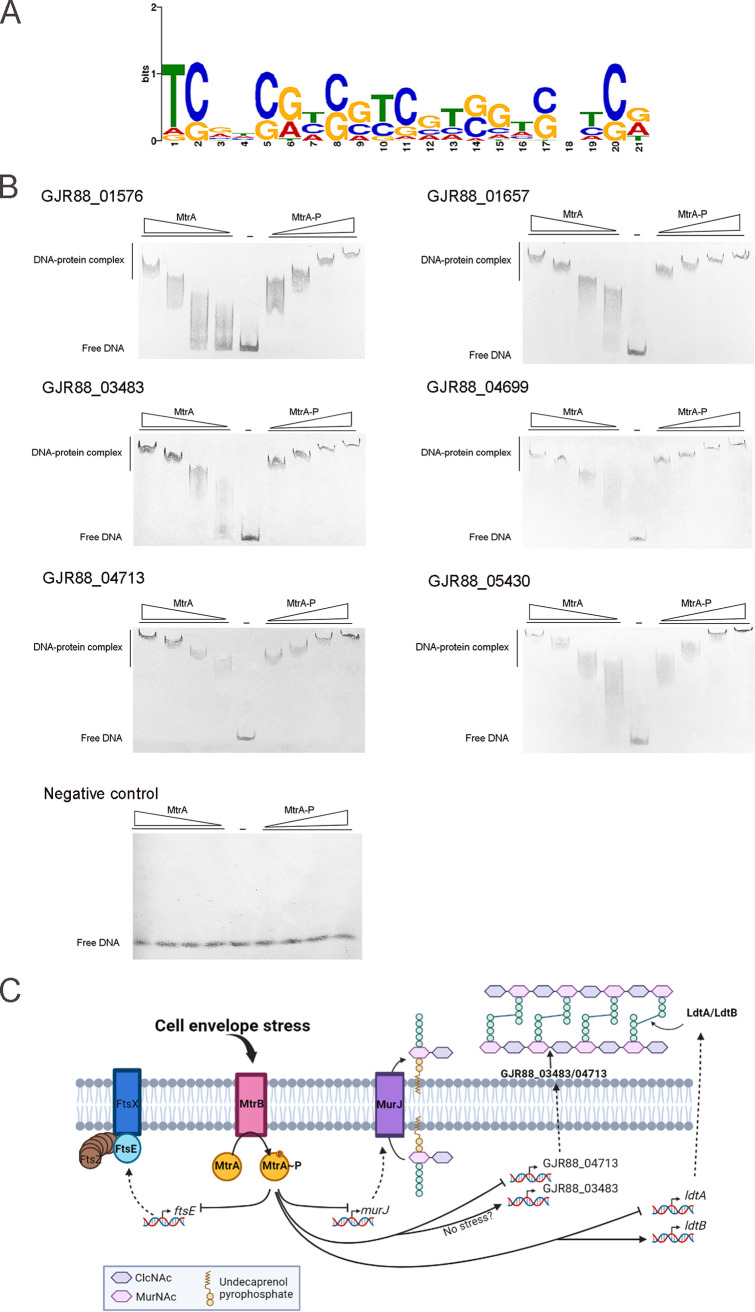
Identification and validation of MtrA motif and analysis of predicted MtrA sites upstream of the genes involved in peptidoglycan homeostasis in *Dietzia* sp. DQ12-45-1b. (A) Predicted consensus MtrA binding site generated from MEME analysis based on CUT&Tag data (48). The *E*-value of the motif is 4.2e-029. The height of each nucleotide represents the relative frequency. (B) EMSA analysis of MtrA binding to the six selected promoters and nonspecific DNA (Escherichia coli DNA fragment). The reactions were performed at different concentrations of MtrA and MtrA-P (0, 0.5, 1, 2.5, and 5 μM). The nonspecific DNA was used as a negative control. (C) Proposed model of MtrA regulatory network on the genes that governed peptidoglycan synthesis and hydrolysis.

To investigate whether the MtrAB TCS directly regulates these genes, we defined the MtrA regulon in *Dietzia* sp. DQ12-45-1b using the Cleavage Under Targets and Tagmentation (CUT&Tag) technology (46). A previous study reported that glutamic acid replacement at a residue corresponding to D56 of MtrA creates a constitutively active MtrA (47). Based on this finding, we generated the MtrA (D56E) mutant and overexpressed this mutant in *Dietzia* sp. DQ12-45-1b wild-type strain for CUT&Tag assay. We analyzed the sequences of the binding regions derived from CUT&Tag data using MEME (48). As shown in Fig. 4A, a 21-nucleotide consensus motif with seven imperfect TCG appears to be essential for MtrA binding (Fig. 4A).

Based on these CUT&Tag data, we further identified an additional 103 DNA-binding genes (Table S2), including 6 genes involved in peptidoglycan biosynthesis (transpeptidase genes *ldtB*, *ldtA*, and flippase gene *murJ*), hydrolysis (hydrolase genes GJR88_03483, GJR88_4713), and cell division (cell division gene *ftsE*). The upstream regions of all these genes contained the TCG-rich consensus motif (Table 2). Moreover, the expression of 5 genes (*ldtB*, *ldtA*, *murJ*, *ftsE*, GJR88_4713) significantly changed in the Δ*mtrAB* mutant compared with the wild-type strain in response to alkaline stresses. In addition, the hydrolase gene GJR88_03483 was downregulated in the Δ*mtrAB* mutant compared to the wild-type strain in pH 8 conditions (Fig. 2, Fig. S4, and Table S1). The transcription levels of these genes were then verified by quantitative reverse transcription-PCR (qRT-PCR) (Fig. S5). We next tested whether MtrA binds to the upstream regions of the above 6 genes using EMSA analysis. As shown in Fig. 4C, MtrA bound to these regions *in vitro* (Fig. 4B). In addition, MtrA failed to bind to the negative control DNA (Fig. 4B). Together, these results demonstrated that MtrA directly represses the expression of *ldtA*, *murJ*, *ftsE*, and GJR88_04713 genes, and activates the *ldtB* and GJR88_03483 genes (Fig. 4C). Critically, the uncontrolled activity of synthetases and hydrolases can lead to an imbalance between peptidoglycan synthesis and degradation, leading to cell deformation and lysis (49, 50). In addition, it has been reported that FtsE promotes divisome assembly and regulates septal peptidoglycan splitting (51). Therefore, MtrA presumably serves both activator and repressor functions critical for maintaining cell envelope homeostasis in *Dietzia* sp. DQ12-45-1b, especially in response to cell envelope stress conditions.

**TABLE 2:** The probes for the MtrA site of the six selected promoters^*a*^

Gene ID	Sequence (5′→3′)	Product	Gene name
GJR88_01576	GTCGGCGCGGTCGGCGTGGTCGTCGTGGGCACGGTCGTCCC	Cell division ATP-binding protein	*ftsE*
GJR88_01657	CTGGCTCAGTTCATGGTCGGAAGGGACATGACGCTCGGAAG	Putative L,D-transpeptidase	*ldtB*
GJR88_03483	GCGTGGGTGATCGTGATGGCGGTGGAGAACGTGGCCGTGCG	Membrane-bound lytic murein transglycosylase B	NA^*b*^
GJR88_04699	ACTGCCGCACTCACCGCGCCCACGGGGTTCTGTTACGTATG	L,D-transpeptidase family protein	*ldtA*
GJR88_04713	TCACAATTCGTCGGTGTGCGACAACCGGGCGGTGGTGGTGT	Lyzozyme M1 (1,4-beta-N-acetylmuramidase)	NA
GJR88_05430	CTCGCCGCGGTCATCACCCTCGTGGTCACCGGTCTGATCCT	Flippase	*murJ*, *mviN*

a The underlined letters of sequences represent the predicted MtrA- binding sites.

b NA, gene name was not assigned.

## DISCUSSION

Here, we demonstrated that the two-component system MtrAB is critical for maintaining normal survival and cell envelope homeostasis in response to various cell envelope stress conditions, such as oxidative, SDS, alkaline, and cell envelope-targeting antibiotic stresses. Through RNA-Seq, CUT&Tag-Seq and EMSA analysis, we found that MtrA directly represses peptidoglycan-related transpeptidase gene *ldtA*, flippase gene *murJ*, hydrolase gene GJR88_04713, cell division gene *ftsE*, and activates transpeptidase gene *ldtB* and hydrolase gene GJR88_03483 in *Dietzia* sp. DQ12-45-1b.

Previous studies have reported that the MtrAB TCS affects tolerance to many antibiotics targeting cell envelope. In Mycobacterium smegmatis, the Δ*mtrA* mutant was found to be more resistant to isoniazid, but more sensitive to vancomycin and rifampin than the parent strain, and showed a modest increase in sensitivity to ampicillin (17). In C. glutamicum, the Δ*mtrAB* mutant was more sensitive to penicillin, vancomycin, and lysozyme, but more resistant to ethambutol compared to the wild-type strain (21). In our study, the Δ*mtrAB* mutant showed significantly higher resistance to vancomycin and decreased resistance to isoniazid, ethambutol, ampicillin, cephalexin, and penicillin compared to *Dietzia* sp. DQ12-45-1b wild-type strain. Isoniazid targets InhA which is a long-chain enoyl-acyl carrier protein reductase essential for the biosynthesis of mycolic acid (52). Ethambutol perturbs the cell wall arabinogalactan biosynthesis (53), while β-lactam antibiotics (i.e., ampicillin, cephalexin, and penicillin) are inhibitors of penicillin-binding proteins (PBPs) (54, 55). Together, these results strongly suggest that the MtrAB TCS plays a critical role in resistance to various antibiotics targeting peptidoglycan in Corynebacterineae. However, we found that the Δ*mtrAB* mutant of *Dietzia* sp. DQ12-45-1b was more resistant to vancomycin. This result differs from earlier findings for M. smegmatis and C. glutamicum. We hypothesize that the resistance to vancomycin of the Δ*mtrAB* mutant in *Dietzia* sp. DQ12-45-1b is caused by the diversity of different bacterial species (Fig. S1 and S2). In addition, vancomycin relies on binding to d-Ala-d-Ala to inhibit the biosynthesis of peptidoglycan (56). d-Ala-d-Ala in the peptidoglycan layers which remain unprocessed by PBPs and the murein monomers in the cytoplasmic membrane are 2 established binding targets for vancomycin. To inhibit nascent peptidoglycan synthesis, vancomycin needs to pass through peptidoglycan layers before binding to murein monomers. As a result, many vancomycin molecules are trapped in the peptidoglycan layers. Previous studies have reported that the thickened cell envelope, the increased non-amidated muropeptide components, and the reduced cross-linking of peptidoglycan contributed to vancomycin resistance in Staphylococcus aureus (VRSA) (57, 58). In *Dietzia* sp. DQ12-45-1b, cell envelope thickness of the Δ*mtrAB* mutant strain was thinner than the wild-type strain under optimal pH conditions (Fig. S6). Based on these findings, we speculate that the Δ*mtrAB* mutant of *Dietzia* sp. DQ12-45-1b possesses a larger number of PBPs unprocessed peptidoglycan or non-amidated muropeptides than wild-type strain to trap more vancomycin molecules. As a result, the Δ*mtrAB* mutant strain exhibits reduced susceptibility to vancomycin. This speculation can be studied in future research. Moreover, our Δ*mtrAB* mutant showed decreased resistance to isoniazid, ethambutol, and β-lactam antibiotics, which further provided evidence for the role of the MtrAB TCS in cell envelope synthesis and metabolism and even the biosynthesis of mycolic acid and arabinogalactan.

It has been reported that peptidoglycan sythetase genes *ftsI*, *dacB1*, *wag31*, and hydrolase genes *ripA*, *rpfA-E* are directly regulated by MtrA in M. tuberculosis (17). Moreover, in C. glutamicum, MtrA directly represses peptidoglycan hydrolase genes *mepA*, *nlpC*, and *rpf2* (23). In our study, we showed that in *Dietzia* sp. DQ12-45-1b, MtrA directly activates peptidoglycan hydrolase gene GJR88_03483, transpeptidase gene *ldtB*, and represses peptidoglycan hydrolase gene GJR88_04713, transpeptidase gene *ldtA*, flippase gene *murJ*, cell division gene *ftsE*. The phylogenetic analysis showed that the MtrA proteins from M. tuberculosis, C. glutamicum, and *Dietzia* sp. DQ12-45-1b are distributed in different clusters in the phylogenetic tree (Fig. S1), which might explain the variations in the genes that MtrA regulated among these strains. These results also suggested that the MtrAB TCS has more diverse functions than what we have revealed.

Many TCSs are involved in sensing signals and regulating adaptive genes for stress tolerance; however, the precise molecular nature of the specific signal involved remains unknown. The specific inductive signals of histidine kinases may correlate with regulatory pathways of response regulators occasionally. For example, the signals for the CpxAR TCS are thought to be secreted, misfolded proteins in the periplasm, with its regulon consisting of genes that encoded protein folding and degradation factors (4, 9, 10, 59). The RcsBCDF system senses lipopolysaccharide (LPS) defects or cell envelope damage, which then causes changes in the expression of genes involved in virulence and capsule biosynthesis (11–13, 60–62). The signal for the WalKR TCS is thought to be the level and/or availability of Lipid II outside the cytoplasmic membrane, with its regulon containing genes involved in cell envelope homeostasis (14, 63). However, the signals which activate the MtrAB TCS in Actinobacteria were unclear. In our study, in *Dietzia* sp. DQ12-45-1b, we found that the MtrAB TCS plays an important role under various cell envelope stresses; as a result, inductive signals for the MtrB sensor presumably were components triggered by multiple stresses such as peptidoglycan fragments. Interestingly, previous studies have reported that peptidoglycan fragments might act as a signaling molecule of Ser/Thr protein kinases (64–66). The signal-induced MtrB may quickly activate MtrA to regulate downstream genes involved in cell division and cell envelope homeostasis. Future research should focus on identifying the precise signals recognized by MtrAB TCS and how MtrB is activated.

Certain peptidoglycan enzymes with overlapping activity can be preferentially used for survival under specific environmental conditions. For example, in Escherichia coli, PBP1a synthetase and MltG hydrolase are required for survival at alkaline pH stress, in addition, PBP1b synthetase and MltA hydrolase are needed under acidic pH (67, 68). In *Dietzia* sp. DQ12-45-1b, both GJR88_03483, and GJR88_04713 directly regulated by MtrA are annotated as peptidoglycan hydrolases with lysozyme activity. This activity allows the cutting of the β-1-4 glycosidic bond between N-acetylmuramic acid and *N*-acetylglucosamine residues (69). Our transcriptome analysis showed that only GJR88_04713 was downregulated in the wild-type strain, but was upregulated in the Δ*mtrAB* mutant at pH 10. However, GJR88_03483 was repressed in the Δ*mtrAB* mutant at optimal pH (pH 8). Therefore, we speculate that GJR88_03483 plays a critical role in pH 8. In addition, GJR88_04713 was required at alkaline pH in *Dietzia* sp. DQ12-45-1b. Except for the different expression levels of GJR88_04713 and GJR88_03483 genes, we also found that in *Dietzia* sp. DQ12-45-1b, LdtA was repressed under alkaline stress, while LdtB was directly activated by MtrA. Previous studies reported that 3–3 cross-links between muropeptides were produced by LD-transpeptidases (LDTs) in bacteria (70, 71). In addition, LDTs can catalyze the formation of the linkage between the Braun lipoprotein (Lpp) and muropeptide in E. coli (72). Multiple functions of LDTs suggested that LdtA and LdtB may contain extra functions in addition to forming the 3–3 cross-links in *Dietzia* sp. DQ12-45-1b in alkaline stress.

In summary, our study provides compelling evidence that in *Dietzia* sp. DQ12-45-1b, the MtrAB TCS fulfills an important role in response to cell envelope stresses and maintaining cell envelope homeostasis, namely, by directly regulating genes involved in peptidoglycan homeostasis and cell division. Because this cell envelope stress sensing and response system might be common in Actinobacteria, our study provides critical insights into the industrial production of applied bacteria and virulence regulation of pathogens. Our findings strongly suggest that TCSs act both as a general sensor as well as a global regulator in maintaining cellular homeostasis, especially during times of harsh environmental conditions. The findings provide novel insights into the understanding of mechanisms for microbial adaptation to diverse environmental stress conditions.

## MATERIALS AND METHODS

### Bacterial strains and growth conditions.

The strains and plasmids used in this study are shown in Table 3.

**TABLE 3:** Strains and plasmids used in this study

Strain or plasmid	Genotype and description	Source and reference
Strains		
*Dietzia* sp. DQ12-45-1b (CGMCC 1.10709)	Wild-type	(32)
DQ12-45-1b/pNV18	Wild-type harboring pNV18; Km^r^	This study
DQ12-45-1bΔ*mtrAB*	DQ12-45-1b *mtrAB* deletion mutant; Sm^r^	This study
DQ12-45-1bΔ*mtrAB/*pNV18	DQ12-45-1b *mtrAB* deletion mutant harboring pNV18; Km^r^ and Sm^r^	This study
DQ12-45-1bΔ*mtrAB*/pNV18-*mtrAB*	DQ12-45-1bΔ*mtrAB* harboring pNV18-*mtrAB*; Km^r^ and Sm^r^	This study
DQ12-45-1b wild/pNV18-*mtrA_D56E_*	DQ12-45-1b wild-type harboring pNV18- *mtrA_D56E_*; Km^r^	This study
E. coli DH5α	Cloning strain	TransGen Biotech, China
Plasmids		
pJV53	*Dietzia*-*E.coli* shuttle vector (acetamide-inducible promoter); Km^r^	(77)
pNV18-DsRed	*Dietzia*-*E.coli* shuttle vector (p45 promoter); Km^r^	(45)
pNV18	Cloned from pNV18-DsRed.T4 without DsRed; Km^r^	This study
pNV18-*mtrAB*	pNV18 + *mtrA* and *mtrB* from DQ12-45-1b; Km^r^	This study
pNV18-*mtrA*	pNV18 + *mtrA* from DQ12-45-1b; Km^r^	This study
pNV18-*mtrA_D56E_*	pNV18 + *mtrA*(phosphorylation-mimic) from DQ12-45-1b; Km^r^	This study

*Dietzia* sp. DQ12-45-1b wild-type, mutant, and complementary strains were cultured in GPY medium (10 g/L glucose, 10 g/L tryptone, and 5 g/L yeast extract) with the appropriate antibiotics (streptomycin 30 μg/mL, and kanamycin 50 μg/mL) at 30°C and shaken at 150 rpm. *E.coli* DH5α and its recombinants were grown inLB medium (10 g/L NaCl, 10 g/L tryptone, and 5 g/L yeast extract) at 37°C with shaking at 150 rpm.

For all growth experiments for *Dietzia* sp. DQ12-45-1b strains under different stress conditions, 96-well plates were used at 30°C with shaking at 600 rpm. Growth curves were monitored at intervals of 24 h via a microplate reader (SpectraMax i3, Molecular Devices) at 600 nm. All the cultures were normalized OD_600_ to 0.1 and were allowed to grow in GPY medium with different stresses. For oxidative, lysozyme, and SDS stress experiments, either 2 μL of 2.5% H_2_O_2_, or 2 mg/mL lysozyme, or 0.5% SDS was added to 200 μL fresh GPY medium, respectively. For osmotic stress experiments, GPY medium containing 5 M NaCl was diluted to a final concentration of 0.625 M by adding fresh GPY medium. For the alkaline pH stress experiment, GPY medium was added with a mixture of 0.2 M Na_2_CO_3_ and 0.2 M NaHCO_3_ to pH 10. All growth curve experiments were performed in 3 biological replicates.

### Phylogenetic analysis.

The amino acid sequences of MtrA and MtrB were obtained from the UniProtKB and NCBI's non-redundant protein (NR) databases (Table S1). The sequences were aligned using the ClustalW (73). Phylogenetic trees were generated using the neighbor-joining method (74) by MEGA software using the default parameters (75).

### Construction of Δ*mtrAB* mutant and complementary strains.

To prepare the electrocompetent cells of *Dietzia* sp. DQ12-45-1b wild/pJV53 (76, 77), *Dietzia* sp. DQ12-45-1b wild/pJV53 was cultured in GPY medium at OD_600_ of 0.05 in the presence of kanamycin (50 μg/mL) and 0.5% glycine overnight at 30°C with shaking at 150 rpm. When grown to OD_600_ of 0.4–0.6, the cultures were added with 0.2% acetamide, 0.2% Tween 80, isoniazid (0.8 mg/mL), and penicillin (0.5 μg/mL). After 4 h of growth at 30°C, cells were harvested and washed twice with 10% glycerin by centrifugation at 1,500g (Eppendorf Centrifuge 5810 R, rotor F-34-6-38) for 10 min at 4°C. The electrocompetent cells were resuspended in 10% glycerin to achieve a final OD_600_ of 50–100, and stored at −80°C for further experiments.

To generate the Δ*mtrAB* mutant strain, we used the double homologous recombination method (77). The upstream and downstream homologous regions of *mtrAB* (~500 bp) were amplified by PCR from the genomic DNA of *Dietzia* sp. DQ12-45-1b using the primers LF/LR and RF/RR, respectively (Table 4). The primers smF and smR were used to amplify the Sm^r^ cassette via PCR. The DNA substrate for the replacement of the *mtrAB* gene was generated by cloning upstream and downstream homologous fragments of *mtrAB* flanking the Sm^r^ cassette using the primers fuF and fuR via fusion PCR (Table 4). The fusion fragment was electroporated into *Dietzia* sp. DQ12-45-1b wild/pJV53 competent cells (78) (Fig. S7). Δ*mtrAB* mutant strain was examined by PCR using the primers idF and idR (Table 4).

**TABLE 4:** Primers used in this study

Primer	Sequence (5′→3′)	Application
LF	TGCTGTTCGCCCTGGACC	Δ*mtrAB* mutant strain
LR	CCTTCATCCGTTTCCACGGTTCGTCGTCGACGACGAGGA	
RF	AAGCGTGCATAATAAGCCCTCTCGACCACTCGGAGTCCAA	
RR	ACCGGACGGAGAGAAGGTGT	
smF	ACCGTGGAAACGGATGAAGG	
smR	AGGGCTTATTATGCACGCTT	
fuF	GTCCTGCTCGACCGCTACGT	
fuR	CAGCACGGTGGCGCAGATCG	
comF	CCCAAGCTTCGACATACACGCCGACACAA	Complementary plasmid
comR	CCCAAGCTTTCACGAACGGACCTCCTCGG	
mtrAF	CCCAAGCTTCGACATACACGCCGACACAA	MtrA phosphorylation-mimic vector
mtrAR	CCCAAGCTTTCACGAGACCGGACCGGCCT	
pNVF	CCCAAGCTTGGCACTGGCCGTCG	
pNVR	CCCAAGCTTGTGCCCGCTGAACTTCTTC	
mtrAD56EF	CCTGCTGGAGCTCATGCTCC	
mtrAD56ER	CGAGACCGGACCGGCCTTGTAC	
pNVD56EF	GTACAAGGCCGGTCCGGTCTCG	
pNVD56ER	GGAGCATGAGCTCCAGCAGG	
1576DIGF	ACCCCCCCGACGGAACCGGT (5′-Digoxigenin)	EMSA
1576DIGR	ATGGGAAGCACTGATCACAC	
1657DIGF	GCTCTGCCAATTGAGCTAAT (5′-Digoxigenin)	
1657DIGR	TAATGGCCCCTTCCGAGCGT	
3483DIGF	CGGCCGCGGATCAGCCGAGA (5′-Digoxigenin)	
3483DIGR	AGCGCAGCCGCGGAGGGGGC	
4699DIGF	CTCTCCGATCGGGCCCCCTC (5′-Digoxigenin)	
4699DIGR	CGTTCTCCTTCGGGCCGCGG	
4713DIGF	GGAACGGAGGGATTTCTTCC (5′-Digoxigenin)	
4713DIGR	GGGGGAAGTATCAAGTAGTA	
5430DIGF	CGCGGTCATCACCCTCGTGG (5′-Digoxigenin)	
5430DIGR	CCGTAGACCTCCTCGACGAC	
16sRTF	GTCTCATGTTGCCAGCACGTT	Real-time PCR
16sRTR	GCAGCCCTCTGTACTAGCCAT	
1576RTF	GGGCGAATTCGCATTTCTCATC	
1576RTR	GGAGGCGGAAGTCCTGAAAG	
1657RTF	TGATCGACGTCTCCGTGGAGGA	
1657RTR	TGTACTCGCGGCTGTACCCA	
3483RTF	TGGGCTCAGGGGATCAACGA	
3483RTR	TAGCCGTCGTCGTTCAGGGT	
4699RTF	CGTCATGATCCGGTTCGACC	
4699RTR	TGCGGAAGGTGTTCTCCTGC	
4713RTF	CCTTCTCGCCGTCGTCATCT	
4713RTR	TCGGCCTCGTAGTAGCGGTT	
5430RTF	GGTCCTGTACGCGCTTCTGA	
5430RTR	GAACTCACCGACGATCCGTC	

For complementary vector construction, the DNA fragment of *mtrA* and *mtrB* genes was cloned from *Dietzia* sp. DQ12-45-1b genomic DNA using the primers comF and comR, both of which contained the HindIII restriction sites. Simultaneously, a DNA fragment containing p45 promoter and HindIII restriction sites was amplified from the pNV18-DsRed plasmid using the primers pF and pR by PCR (Table 4). The HindIII-digested DNA fragment of *mtrAB* was ligated into the HindIII-digested linear fragment from pNV18-DsRed without the *dsRed* gene. To obtain the complementary strain, the recombinant plasmid pNV18-*mtrAB* was electroporated into *Dietzia* sp. DQ12-45-1b Δ*mtrAB* mutant electrocompetent cells.

### Construction of *mtrA* phosphorylation-mimic vector.

The D56E mutation at the phosphorylation site in response regulator MtrA which mimics the aspartate-phosphoryl state and bypassed the requirements for MtrB sensor kinase has been already verified (47). The complete *mtrA* gene was amplified from the genomic DNA of *Dietzia* sp. DQ12-45-1b using the primers mtrAF and mtrAR with the HindIII restriction sites on both sides. The DNA fragment of the vector pNV18 was amplified with the HindIII restriction sites using the primers pNVF and pNVR from the pNV18-DsRed plasmid (Table 4). Next, the HindIII-digested DNA fragment of *mtrA* was ligated into the plasmid fragment to obtain plasmid pNV18-*mtrA*. Then the primers mtrAD56EF, mtrAD56ER, and pNVD56EF, pNVD56ER were used to amplify by PCR from pNV18-*mtrA* plasmid to replace the aspartic acid at the 56th codon with glutamate to create plasmid pNV18-*mtrA_D56E_* using the Hieff Clone Plus One Step Cloning Kit (Yeasen). The recombinant plasmid pNV18-*mtrA_D56E_* was electroporated into *Dietzia* sp. DQ12-45-1b wild competent cells.

### Antibiotics susceptibility tests.

The micro-broth dilution method was used to determine the MICs of the various antibiotics for *Dietzia* sp. DQ12-45-1b strains in 96-well plates (79). Serial 2-fold dilutions of tested antibiotics were done in the GPY medium with kanamycin (50 μg/mL). When grown to the middle exponential phase, cells were harvested, and the pellets were washed twice with PBS buffer (pH 7.4) by centrifugation at 5,000 rpm (Eppendorf Centrifuge 5417 R, rotor FA-45-24-11) for 5 min at 4°C. All the cultures were normalized OD_600_ to 0.05 with different antibiotic stresses per well at 30°C with shaking at 600 rpm for 24 h. Cultures without antibiotic stress were regarded as control. The concentration of antibiotics at which bacterial growth was inhibited by 90% compared to the control wells was MIC we tested.

### Bright-field microscopy.

For cell morphologic observation, cells were collected and washed three times by PBS buffer (pH 7.4) by centrifugation at 5,000 rpm (Eppendorf Centrifuge 5417 R, rotor FA-45-24-11) for 5 min at 4°C. The microscopy images were examined under a 100× oil immersion lens with a bright-field microscope. The cell length quantitative measurement uses the software Digimizer, MedCalc Ltd (80). An *unpaired t test* was used for statistical analysis.

### CUT&Tag-Seq.

CUT&Tag assay was performed in Ruiyuan Biotechnology Co., Ltd. (Nanjing, China). Cells overexpressing MtrA_D56E_ were grown to the middle exponential phase and then collected by centrifugation at 5,000 rpm (Eppendorf Centrifuge 5417 R, rotor FA-45-24-11) for 10 min at 4°C. The cells were digested at 37°C using 100 mg/mL lysozyme for 24 h, then collected by centrifugation at 5,000 rpm (Eppendorf Centrifuge 5417 R, rotor FA-45-24-11) for 10 min at 4°C. For CUT&Tag and library amplification, Hyperactive In-Situ ChIP Library Prep Kit for Illumina (TD901, Vazyme) was performed according to the manufacturer’s instructions. Briefly, cells were incubated with 10 μL pre-treated ConA beads at room temperature for 10 min. Next, 50 μL pre-cooled antibody buffer with 0.5 μg His-tag antibody was added to the collected reaction solution for incubation at room temperature for 2 h. After primary antibody incubation, the collected reaction solution was incubated with 50 μL dig-wash buffer with 0.5 μg secondary antibody at room temperature for 1 h. Next, the collected reaction solution was incubated at room temperature for 1 h with 100 μL dig-300 buffer containing 2 μL hyperactive pG-Tn5 transposon. Subsequently, 300 μL tagmentation buffer was added to the collected reaction solution for fragmentation at 37°C for 1 h. The reaction was stopped by adding 2.5 μL 20 mg/mL Proteinase K, 3 μL 10% SDS, and 10 μL 0.5M EDTA at 50°C for 1 h. Lastly, DNA was extracted by phenol-chloroform and ethanol precipitation. PCR was used for the subsequent library amplification. Sequencing was performed using the Illumina Novaseq 6000 (Novogene).

### Electrophoretic mobility shift assay.

DNA fragments were amplified using Digoxigenin (DIG)-labeled primers as listed in Table 4. MtrA protein was phosphorylated by EnvZ (47). Next, 10 pM of purified DNA fragments were incubated using 0–5 μM MtrA or MtrA-P in binding buffer containing 5 mM Tris-HCl (pH 7.6), 50 mM KCl, 0.5 mM EDTA, 5 mM (NH_4_)_2_SO_4_, 0.5 mM DTT, 0.1% Tween 20, 5 mM MgCl_2_, and 5 mM CaCl_2_ at room temperature for 30 min. The reaction was stopped with 10×loading buffer (50 mM Tris-HCl (pH 6.8), 30% glycerol, 0.12% bromophenol blue). The reaction mixtures were resolved in 5% nondenaturing polyacrylamide gels for 3 h at 40 V. Next, the DNA and proteins were transferred to nylon membranes using the semi-dry transfer method for 10 min at 10 V. For the subsequent immunological detection, DIG High Prime DNA Labeling and Detection Starter Kit I (Roche) was performed following manufacturer’s instructions.

### Transcriptome analysis.

RNA was isolated using cells grown to the mid-exponential phase. RNA sequencing was performed using the Illumina NovaSeq6000 platform (Magigene Ltd.). Fold changes of selected genes were calculated as a ratio of treatment and control groups via fragments per transcript kilobase per million fragments mapped (FPKM) values. The differential expression analysis was performed using the DESeq2 software (81). The resulting *P*-values were adjusted using the Benjamini-Hochberg method for controlling the false discovery rate (FDR) (82). The differentially expressed genes (DEGs) were defined with a threshold of fold change ≥ 2 and false discovery rate (FDR) ≤0.05. Heatmaps were produced using MeV software. The KEGG Orthology (KO) assignment was obtained using the bi-directional best hit (BBH) method by the online KEGG Automatic Annotation Server (KAAS) (http://www.genome.jp/kegg/kaas/). Enrichment analysis of DEGs in the KEGG pathways (FDR ≤0.05) was performed using the OmicShare tools (www.omicshare.com/tools). The volcano plot was also produced using the OmicShare tools. Three biological replicates per condition were used in RNA-seq experiments.

### Quantitative reverse transcription-PCR.

The *Dietzia* cells were collected when grown to the mid-exponential phase. Total RNA from the collected cells was extracted and purified using RNAiso Plus reagent (TaKaRa). Reverse transcription was carried out using the ReverTra Ace reverse transcription kit (TOYOBO) and the random primers. The reverse transcription products (cDNA) were used as templates for real-time PCR. Relative quantities of cDNA were normalized for the 16S rRNA gene. Real-time PCR was performed using the primers listed in Table 4 with SYBR green *Ex Taq* kit II (TaKaRa) in the CFX9 real-time system and C1000 thermal cycler (Bio-Rad). The gene expression level was calculated by the 2^-ΔΔCt^ method (83). Moreover, the nuclease-free water in place of cDNA was used to be a negative control. All the experiments were repeated in triplicate.

### Data availability.

The transcriptomic raw sequence has been deposited to the National Microbiology Data Center (https://nmdc.cn/) under accession ID NMDC10018150 (https://nmdc.cn/resource/genomics/project/detail/NMDC10018150).
