# Supplementary material for: The Roles of the Two-Component System, MtrAB, in Response to Diverse Cell Envelope Stresses in Dietzia sp. DQ12-45-1b
Source: Appl Environ Microbiol. 2022 Oct 3;88(20):e01337-22. doi: 10.1128/aem.01337-22 (PMC9599347; doi:10.1128/aem.01337-22)
Supplement: Supplemental file 1 — Supplemental material. Download aem.01337-22-s0001.pdf, PDF file, 1.2 MB [file aem.01337-22-s0001.pdf]

Supplementary Information

The roles of the two-component system MtrAB in response to diverse cell envelope stresses in *Dietzia* sp. DQ12-45-1b

Xiaoyu Qin[1], Yong Nie[1]*, Xiao-Lei Wu[1,2,3]*

[1] College of Engineering, Peking University, Beijing 100871, China

[2] Institute of Ocean Research, Peking University, Beijing 100871, China

[3] Institute of Ecology, Peking University, Beijing 100871, China

*Corresponding author: Research Scientist, College of Engineering, Peking University.

Tel: +86 10-62759047; Fax: +86 10-62759047; E-mail: nieyong@pku.edu.cn

*Corresponding author: Professor, College of Engineering, Peking University.

Tel: +86 10-62759047; Fax: +86 10-62759047; E-mail: xiaolei_wu@pku.edu.cn

Figure S1

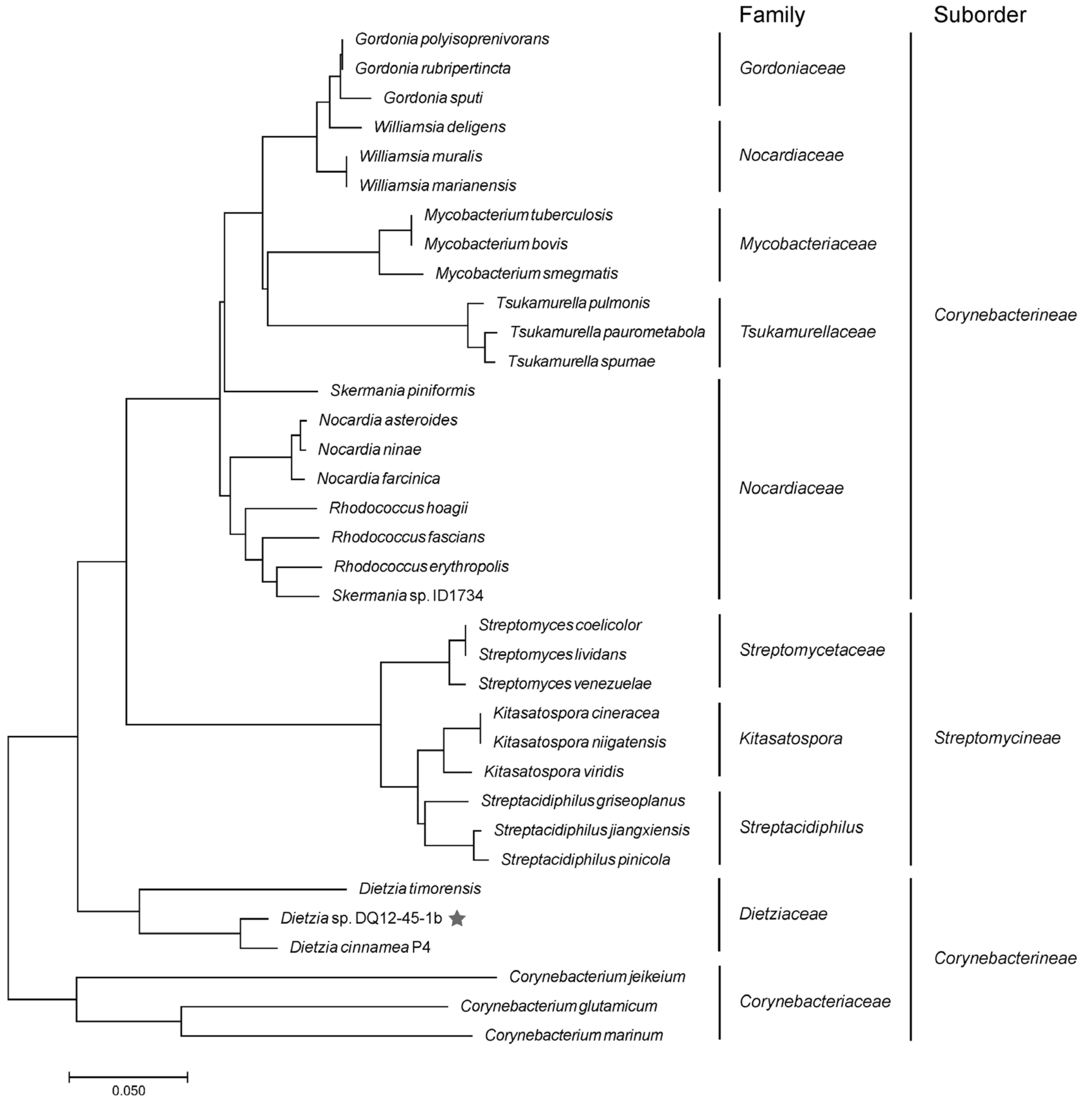

Fig S1. Phylogenetic tree of the amino acid sequence of MtrA of *Dietzia* sp. DQ12-45-1b and other strains of genera classified in the suborders *Corynebacterineae* and *Streptomycineae*. Grey star represents MtrA in this study.

Figure S2

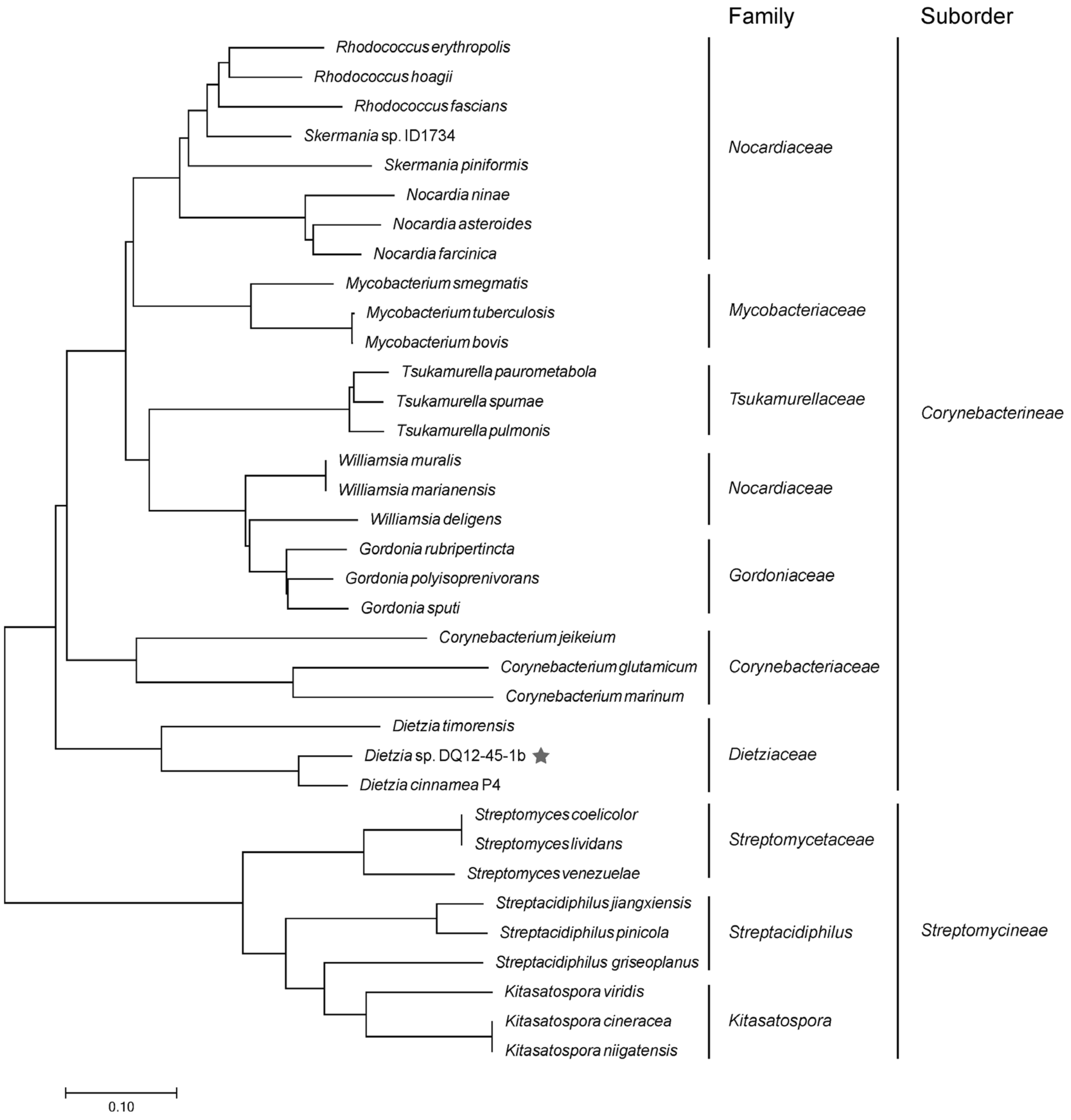

Fig S2. Phylogenetic tree of the amino acid sequence of MtrB of *Dietzia* sp. DQ12-45-1b and other strains of genera classified in the suborders *Corynebacterineae* and *Streptomycineae*. Grey star represents MtrB in this study.

Figure S3

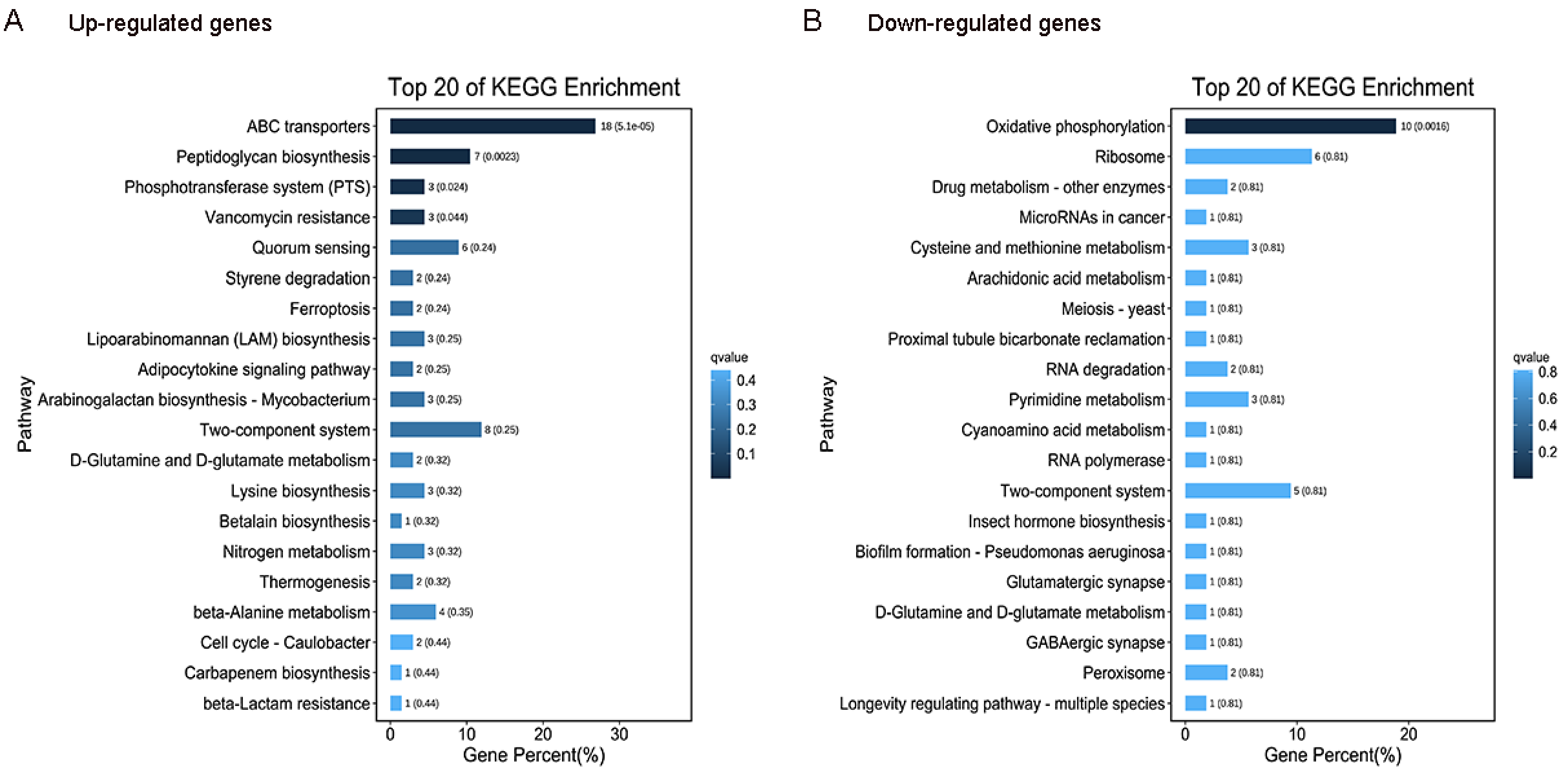

Fig S3. KEGG enrichment analysis. Barplot of KEGG enriched from up-regulated genes (A) and down-regulated genes (B) of the Δ*mtrAB* mutant

compared to the wild-type strains at pH 10.

Figure S4

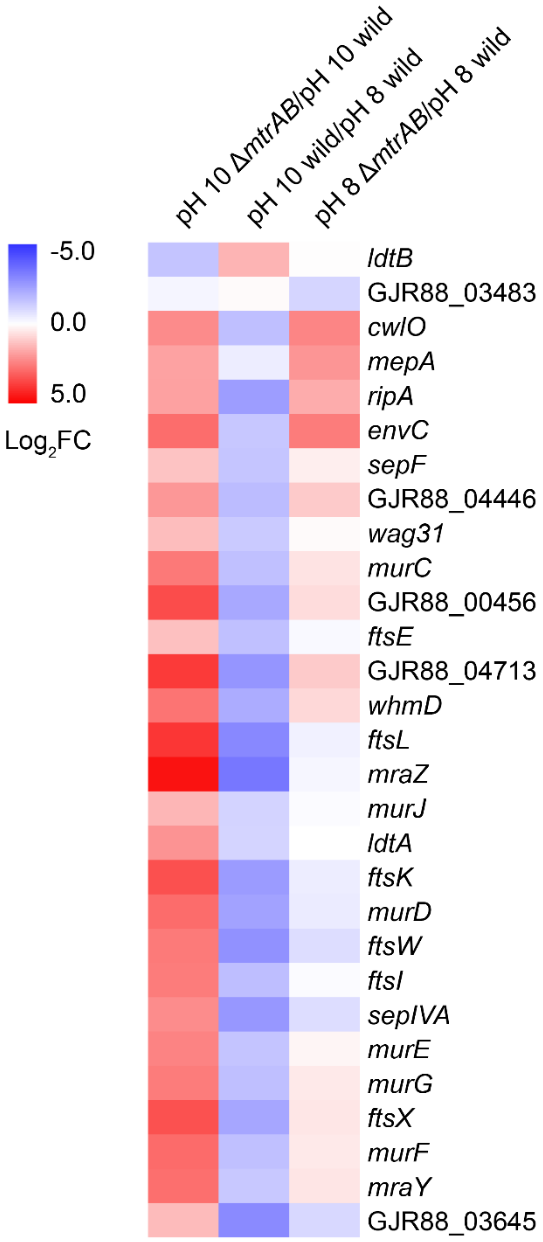

Fig S4. Transcriptomic analysis of the wild-type and Δ*mtrAB* mutant strains under pH 8 and pH 10 conditions. Heatmap of the genes involved in cell envelope homeostasis induced and repressed in the wild-type and Δ*mtrAB* mutant strains under different pH conditions (fold change ⩾ 2, FDR ⩽ 0.05). Red represents up-regulated genes, blue represents down-regulated genes.

Figure S5

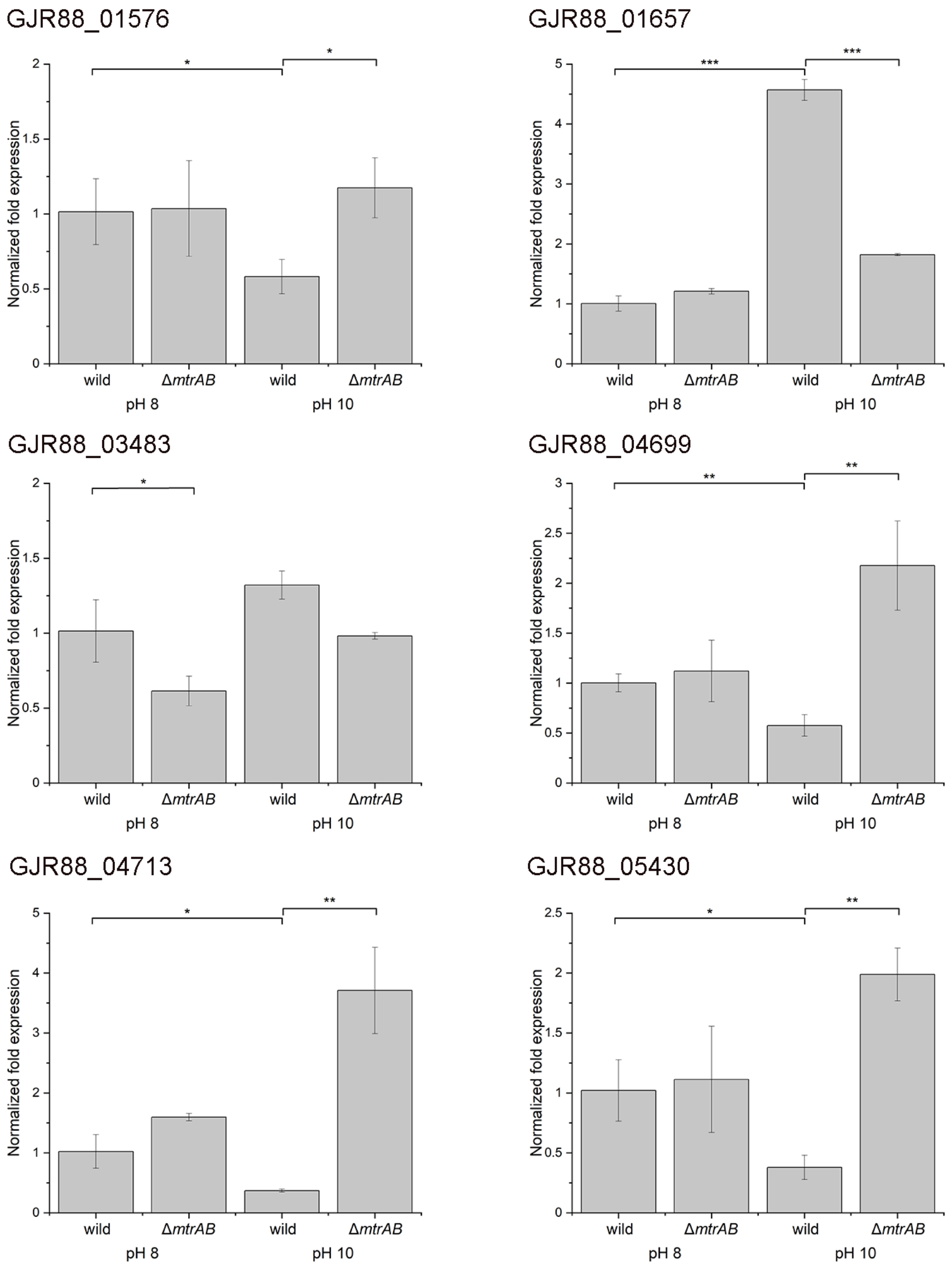

Fig S5. Quantitative reverse transcription PCR (qRT-PCR) analysis of the six selected genes transcription levels in *Dietzia* sp. DQ12-45-1b wild-type and Δ*mtrAB* mutant cells in pH 8 and pH 10 conditions. Error bars represent standard errors (n = 3). Mean values were compared by Student's *t* test. ***, $p < 0.001$; **, $p < 0.01$; *, $p < 0.05$.

Figure S6

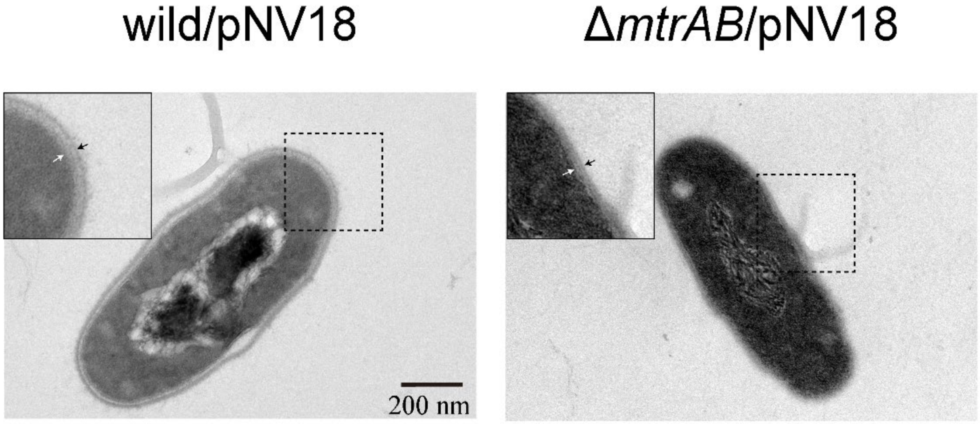

Fig S6. Representative thin-section transmission electron microscopy of the cell envelope of *Dietzia* sp. DQ12-45-1b wild-type and Δ*mtrAB* mutant cells. The distance between black and white arrows represents the thickness of the cell envelope. Inserts show a small portion of cell cross-sections for each image.

Figure S7

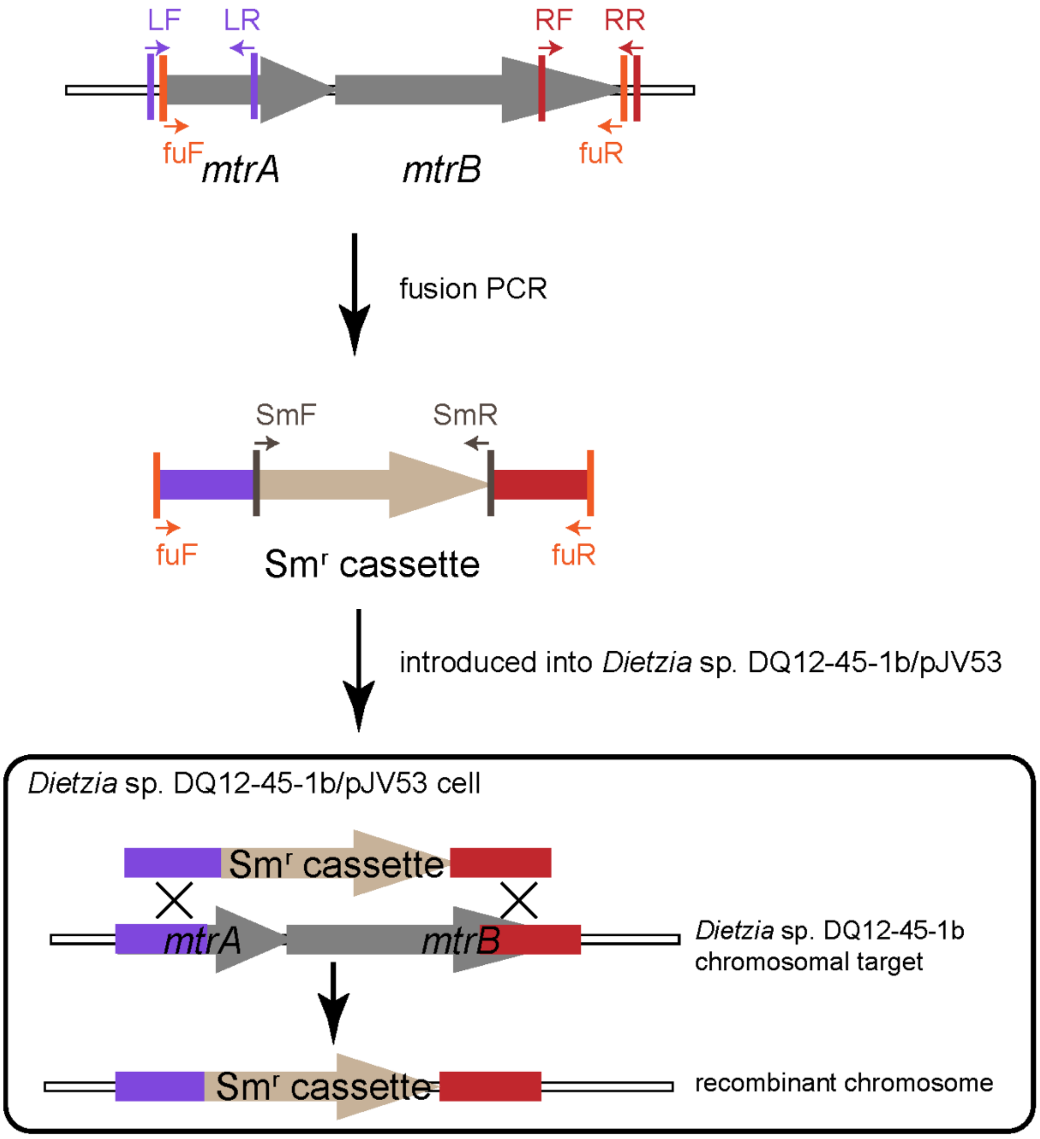

Fig S7. Schematic diagram of the double homologous recombination to construct the Δ*mtrAB* mutant strain. Purple and red boxes represent the upstream and downstream homologous regions of *mtrAB* (~500 bp). Fusion PCR was performed to obtain a DNA fragment which containing the upstream and downstream homologous regions of *mtrAB* flanking the streptomycin cassette. The linear fusion fragment was introduced into *Dietzia* sp. DQ12-45-1b wild/pJV53 competent cells to generate the Δ*mtrAB*

mutant strain.

Table S1

Table S1 The sequences of MtrA and MtrB from different strains of genera classified in the suborders *Corynebacterineae* and *Streptomycineae* with their accession numbers.

Strains	Accession numbers
MtrA	
Corynebacterium glutamicum	A0A1Q6BJJ6
Corynebacterium jeikeium (strain K411)	Q4JTP7
Corynebacterium marinum DSM 44953	A0A0B6TJP8
Dietzia sp. DQ12-45-1b	A0A6I6G9B8
Dietzia cinnamea P4	E6J9Z1
Dietzia timorensis	A0A173LK32
Gordonia polyisoprenivorans (strain DSM 44266 / VH2)	H6N057
Gordonia rubripertincta	A0A222TMX7
Gordonia sputi NBRC 100414	H5U5A6
Mycobacterium tuberculosis (strain ATCC 25618 / H37Rv)	P9WGM7
Mycobacterium smegmatis (strain ATCC 700084 / mc(2)155)	A0QTK2
Mycobacterium bovis (strain ATCC BAA-935 / AF2122/97)	P0A5Z5

Nocardia asteroides NBRC 15531	U5E9D7
Nocardia farcinica	A0A0H5P645
Nocardia ninae NBRC 108245	A0A511MV83
Rhodococcus erythropolis (strain PR4 / NBRC 100887)	C0ZWZ1
Rhodococcus fascians D188	A0A161GI57
Rhodococcus hoagii ATCC 33707	E9T3C0
Skermania sp. ID1734	WP_143771670.1
Skermania piniformis DSM 43998	WP_066472878.1
Williamsia muralis DSM 44343	WP_023955897.1
Williamsia deligens	WP_253647539.1
Williamsia marianensis DSM 44944	PVY34339.1
Tsukamurella paurometabola	A0A3P8K3I2
Tsukamurella spumae	WP_168545325.1
Tsukamurella pulmonis NCTC13230	WP_068568224.1
Streptomyces coelicolor (strain ATCC BAA-471 / A3(2) / M145)	Q9KYW8
Streptomyces lividans 1326	A0A7U9HB84
Streptomyces venezuelae (strain ATCC 10712 / CBS 650.69 / DSM 40230 / JCM 4526 / NBRC 13096 / PD 04745)	F2R521
Kitasatospora viridis	A0A561UK63

Kitasatospora cineracea	A0A8G1XCK5
Kitasatospora niigatensis	A0A3N4RMA1
Streptacidiphilus jiangxiensis	A0A1H7RQL9
Streptacidiphilus pinicola	WP_042373547.1
Streptacidiphilus griseoplanus	WP_055587002.1
MtrB	
Corynebacterium glutamicum	A0A8G0CH27
Corynebacterium jeikeium (strain K411)	Q4JTP8
Corynebacterium marinum DSM 44953	A0A0B6TPI9
Dietzia sp. DQ12-45-1b	A0A6I6GGU6
Dietzia cinnamea P4	E6J9Z2
Dietzia timorensis	A0A173LM73
Gordonia polyisoprenivorans (strain DSM 44266 / VH2)	H6N056
Gordonia rubripertincta	A0A222TNF3
Gordonia sputi NBRC 100414	H5U5A7
Mycobacterium tuberculosis (strain ATCC 25618 / H37Rv)	P9WGK9
Mycobacterium smegmatis (strain ATCC 700084 / mc(2)155)	A0QTK3
Mycobacterium bovis (strain ATCC BAA-935 / AF2122/97)	P59963

Nocardia asteroides NBRC 15531	U5EK50
Nocardia farcinica	A0A0H5P5Q8
Nocardia ninae NBRC 108245	A0A511MU96
Rhodococcus erythropolis (strain PR4 / NBRC 100887)	C0ZWZ2
Rhodococcus fascians D188	A0A165KU94
Rhodococcus hoagii ATCC 33707	E9T3C1
Skermania sp. ID1734	WP_255450366.1
Skermania piniformis DSM 43998	WP_246600180.1
Williamsia muralis DSM 44343	WP_062798810.1
Williamsia deligens	WP_253648004.1
Williamsia marianensis DSM 44944	PVY34338.1
Tsukamurella paurometabola	A0A3P8L3A2
Tsukamurella spumae	WP_246207481.1
Tsukamurella pulmonis NCTC13230	WP_114652146.
Streptomyces coelicolor (strain ATCC BAA-471 / A3(2) / M145)	Q9KYW9
Streptomyces lividans 1326	A0A7U9HBG7
Streptomyces venezuelae (strain ATCC 10712 / CBS 650.69 / DSM 40230 / JCM 4526 / NBRC 13096 / PD 04745)	F2R520
Kitasatospora viridis	A0A561UK57

Kitasatospora cineracea	A0A8G1XBX1
Kitasatospora niigatensis	A0A3N4SDF9
Streptacidiphilus jiangxiensis	A0A1H7RRD8
Streptacidiphilus pinicola	WP_111507558.1
Streptacidiphilus griseoplanus	WP_245687237.1

Table S2

Table S2 Fold-change values of the selected genes involved in cell envelope homeostasis in the Δ*mtrAB* mutant and wild-type strains

Gene ID	Gene name	pH10 wild/pH8 wild		pH10 Δ*mtrAB*/pH10 wild		pH8 Δ*mtrAB*/pH8 wild		pH10 Δ*mtrAB*/ pH8 Δ*mtrAB*		Gene product description
		Log_2FC	FDR	Log_2FC	FDR	Log_2FC	FDR	Log_2FC	FDR	
Peptidoglycan synthesis										
GJR88 _02065	*murE*	-1.45	0.000	2.44	0.000	0.21	0.326	0.79	0.000	UDP-N-acetylmuramoylalanyl-D-glutamate--2,6-diaminopimelate ligase
GJR88 _02066	*murF*	-1.55	0.000	2.93	0.000	0.45	0.021	0.94	0.000	UDP-N-acetylmuramoyl-tripeptide--D-alanyl-D-alanine ligase
GJR88 _02068	*mraY*	-1.34	0.000	2.85	0.000	0.52	0.003	1.00	0.000	phospho-N-acetylmuramoyl-pentapeptide-transferase
GJR88 _02071	*murD*	-2.27	0.000	2.91	0.000	-0.46	0.028	1.10	0.000	UDP-N-acetylmuramoyl-L-alanyl-D-glutamate synthetase
GJR88 _02073	*ftsW*	-2.70	0.000	2.64	0.000	-0.82	0.000	0.75	0.000	Cell division protein
GJR88 _05430	*murJ*	-1.06	0.000	1.43	0.000	-0.10	0.676	0.47	0.022	Lipid II flippase
GJR88 _02075	*murG*	-1.55	0.000	2.60	0.000	0.44	0.083	0.62	0.013	UDP-N-acetylglucosamine--N-acetylmuramyl-

										pyrophosphoryl-undecaprenol N-acetylglucosamine
GJR88 _02077	*murC*	-1.53	0.000	2.65	0.000	0.56	0.003	0.55	0.004	UDP-N-acetylmuramate--L-alanine ligase
GJR88 _02062	*ftsI* (*pbpB*)	-1.58	0.000	2.61	0.000	-0.09	0.723	1.11	0.000	Penicillin-binding protein
GJR88 _01657	*ldtB*	1.49	0.000	-1.44	0.000	0.06	0.825	-0.00	1	Putative L,D-transpeptidase LppS
GJR88 _04699	*ldtA*	-1.04	0.000	2.12	0.000	0.02	0.970	1.06	0.000	L,D-transpeptidase family protein
Peptidoglycan hydrolysis										
GJR88 _02580	*ripA*	-2.39	0.000	1.85	0.000	1.63	0.000	-2.17	0.000	Peptidoglycan endopeptidase
GJR88 _04713	NA	-2.59	0.000	3.89	0.000	1.06	0.000	0.25	0.473	1,4-beta-N-acetylmuramidase
GJR88 _02028	*cwlO*	-1.55	0.000	2.28	0.000	2.41	0.000	-1.68	0.000	Peptidoglycan endopeptidase
GJR88 _00456	NA	-2.13	0.000	3.54	0.000	0.70	0.001	0.71	0.001	N-acetylmuramoyl-L-alanine amidase
GJR88 _03658	*mepA*	-0.41	0.125	1.84	0.000	2.09	0.000	-0.67	0.006	Murein DD-endopeptidase
GJR88 _RS174 45	NA	-1.23	0.000	0.82	0.000	0.40	0.059	-0.81	0.000	M23 peptidase domain-containing protein
GJR88 _03129	NA	-1.31	0.000	0.79	0.009	0.93	0.001	-1.45	0.000	Membrane-bound lytic murein transglycosylase

GJR88_03483	NA	0.10	0.686	-0.23	0.361	-1.01	0.000	0.89	0.000	Membrane-bound lytic murein transglycosylase B
Cell division										
GJR88_02816	*ftsK*	-2.44	0.000	3.47	0.000	-0.43	0.032	1.45	0.000	Cell division protein
GJR88_01576	*ftsE*	-1.55	0.000	1.24	0.000	-0.13	0.604	-0.17	0.537	Cell division ATP-binding protein
GJR88_01577	*ftsX*	-2.16	0.000	3.45	0.000	0.49	0.054	0.79	0.001	Cell division protein
GJR88_02061	*ftsL*	-2.91	0.000	3.98	0.000	-0.32	0.137	1.39	0.000	Cell division protein
GJR88_02085	*sepF*	-1.41	0.000	1.18	0.000	0.34	0.108	-0.56	0.006	Cell division protein
GJR88_02952	*sepIVA*	-2.54	0.000	2.27	0.000	-0.81	0.000	0.54	0.009	Cell division protein
Related transcriptional regulators										
GJR88_02058	*mraZ*	-3.33	0.000	4.68	0.000	-0.21	0.439	1.56	0.000	Transcriptional regulator
GJR88_02225	*envC*	-1.36	0.000	2.90	0.000	2.61	0.000	-1.07	0.000	Murein hydrolase activator
GJR88_04446	NA	-1.64	0.000	2.06	0.000	1.04	0.000	-0.63	0.016	LytR cell envelope-related transcriptional attenuator
GJR88_01472	*whiB2* (*whmD*)	-2.03	0.000	2.75	0.000	0.77	0.001	-0.05	0.881	WhiB family transcriptional regulator
GJR88_03645	NA	-2.86	0.000	1.34	0.000	-0.93	0.002	-0.59	0.077	Putative AsnC family transcriptional regulator

Table S3

Table S3 MtrA regulon in *Dietzia* sp. DQ12-45-1b

Gene ID	Functional group	Product	Gene name	Distance of ChIP peak from start codon
GJR88_00635	Amino acids biosynthesis and metabolism	4-aminobutyrate aminotransferase and related aminotransferases	*toa*	-384
GJR88_04752	Amino acids biosynthesis and metabolism	arginine biosynthesis bifunctional protein ArgJ	*argJ*	138
GJR88_00830	Carbon metabolism	putative 2-oxoglutarate--ferredoxin oxidoreductase alpha subunit	*korA*, *oorA*, *oforA*	181
GJR88_01984	Carbon metabolism	pyruvate dehydrogenase complex E2, dihydrolipoamide acetyltransferase	DLST, *sucB*	66
GJR88_03291	Carbon metabolism	putative enoyl-CoA hydratase/isomerase family protein	*crt*	95
GJR88_03448	Carbon metabolism	enolase	ENO, *eno*	465
GJR88_00899	Cofactors biosynthesis and metabolism	dihydropteroate synthase	*folP*	-127
GJR88_01262	Cofactors biosynthesis and metabolism	bifunctional uroporphyrinogen-III synthetase/response regulator domain protein	*hemD*, UROS	-224

GJR88_04465	Cofactors biosynthesis and metabolism	thiazole synthase	*thiG*	119
GJR88_00816	Flp pilus assembly	Flp pilus assembly protein	*tadB*	-171
GJR88_00457	Galactose metabolism	UDP-galactopyranose mutase	*glf*	-166
GJR88_02457	Lipid biosynthesis and metabolism	putative cyclopropane fatty acid synthase	*cfa*	-138
GJR88_02672	Nucleotide metabolism	orotidine 5'-phosphate decarboxylase	*pyrF*	264
GJR88_02456	Oxidative phosphorylation	NADH dehydrogenase	*ndh*	88
GJR88_05170	Oxidative phosphorylation	NADH/ubiquinone/plastoquinone (complex I)	*ndhF*	188
GJR88_01576	Peptidoglycan biosynthesis and metabolism	cell division ATP-binding protein FtsE	*ftsE*	-253
GJR88_01657	Peptidoglycan biosynthesis and metabolism	Putative L,D-transpeptidase LppS	*ldtB*	-85
GJR88_03483	Peptidoglycan biosynthesis and metabolism	Membrane-bound lytic murein transglycosylase B	_	99
GJR88_04699	Peptidoglycan biosynthesis and metabolism	L,D-transpeptidase family protein	*ldtA*	-132
GJR88_04713	Peptidoglycan biosynthesis and metabolism	Lyzozyme M1 (1,4-beta-N-acetylmuramidase)	_	-27
GJR88_05430	Peptidoglycan biosynthesis and metabolism	flippase	*murJ*, *mviN*	-193
GJR88_04164	Phenylalanine metabolism	phenylacetate--CoA ligase	*paaK*	39

GJR88_03900	Protein export	preprotein translocase subunit SecE	*secE*	158
GJR88_00203	Regulator	Lsr2-like protein	_	2
GJR88_00364	Regulator	transcriptional regulator	_	-258
GJR88_00530	Regulator	ArsR family transcriptional regulator	_	-212
GJR88_00595	Regulator	two component system response regulator	_	181
GJR88_03511	Regulator	putative TetR family transcriptional regulator	_	83
GJR88_04029	Regulator	TetR/AcrR family transcriptional regulator	_	-38
GJR88_04146	Regulator	protein kinase/LuxR family transcriptional regulator	_	240
GJR88_04241	Regulator	transcriptional regulator	*cynR*	-71
GJR88_04269	Regulator	transcriptional regulator, Cro/CI family	_	133
GJR88_05073	Regulator	TetR family transcriptional regulator	_	54
GJR88_05122	Regulator	transcriptional regulator, TetR family protein		207
GJR88_00004	Replication and repair	DNA polymerase III subunit beta	DPO3B, *dnaN*	-413
GJR88_00598	Replication and repair	DNA polymerase III, gamma/tau subunits	DPO3G, *dnaX*	-210
GJR88_03430	Replication and repair	transposase IS4 family protein	_	-538
GJR88_02834	RNA degradation	polynucleotide phosphorylase/polyadenylase	*pnp*, PNPT1	293
GJR88_02601	Secondary metabolites biosynthesis	lycopene beta-cyclase	*lcyB*, *crtL1*, *crtY*	-231
GJR88_04350	Secondary metabolites biosynthesis	limonene-1,2-epoxide hydrolase	E3.3.2.8	-97
GJR88_04119	Stress response	Asp23/Gls24 family envelope stress response	_	43

		protein		
GJR88_04560	Stress response	ATP-dependent Clp protease ATP-binding subunit	*clpB*	92
GJR88_04685	Stress response	GlsB/YeaQ/YmgE family stress response membrane protein	_	-40
GJR88_05436	Stress response	thioredoxin reductase	*trxB*	-1
GJR88_04555	Sulfur metabolism	3-mercaptopyruvate sulfurtransferase	TST, MPST, *sseA*	361
GJR88_01343	Transcription	RNA polymerase sigma factor SigD	SIG3.2, *rpoE*	-290
GJR88_01580	Translation	SsrA-binding protein	*smpB*	-101
GJR88_02314	Translation	50S ribosomal protein L35	RP-L35, MRPL35, *rpmI*	-104
GJR88_03780	Translation	50S ribosomal protein L2	RP-L2, MRPL2, *rplB*	51
GJR88_03390	Transporter	iron-siderophore ABC transporter substrate-binding protein	ABC.FEV.S	-85
GJR88_03923	Transporter	molybdenum ABC transporter, periplasmic molybdate-binding protein	*modA*	-69
GJR88_04181	Transporter	Branched-chain amino acid ABC-type transport system, permease components	*livH*	163
GJR88_04239	Transporter	citrate transporter, CitMHS family	TC.CITMHS	32
GJR88_04270	Transporter	branched-chain amino acid permease	_	-142

GJR88_05074	Transporter	drug/metabolite transporter (DMT) superfamily transporter	_	-47
GJR88_RS17255	Unknown	hypothetical protein	_	-357
GJR88_00194	Unknown	hypothetical protein	_	-30
GJR88_00391	Unknown	hypothetical protein	_	164
GJR88_00393	Unknown	hypothetical protein	_	-16
GJR88_00398	Unknown	hypothetical protein	_	-41
GJR88_00406	Unknown	hypothetical protein	_	-97
GJR88_00440	Unknown	hypothetical protein	_	37
GJR88_00647	Unknown	trypsin-like serine protease	_	77
GJR88_00676	Unknown	TPR repeat protein	_	-275
GJR88_00693	Unknown	hypothetical protein	_	-67
GJR88_00703	Unknown	hypothetical protein	_	-253
GJR88_00728	Unknown	CAP domain-containing protein	_	206
GJR88_01268	Unknown	hypothetical protein	_	70
GJR88_01366	Unknown	hypothetical protein	_	22
GJR88_01367	Unknown	sucrase ferredoxin	_	-51
GJR88_01694	Unknown	hypothetical protein	_	-39
GJR88_01761	Unknown	trigger factor	*tig*	-205

GJR88_01868	Unknown	YdcF family protein	_	-48
GJR88_02038	Unknown	3'-5' exoribonuclease	_	-18
GJR88_02129	Unknown	NUDIX hydrolase	_	82
GJR88_02167	Unknown	hypothetical protein	_	322
GJR88_02228	Unknown	mycothiol-dependent formaldehyde dehydrogenase	E1.1.1.306	-192
GJR88_02383	Unknown	bifunctional nuclease family protein	K08999	-9
GJR88_02461	Unknown	Acyl dehydratase	_	22
GJR88_02462	Unknown	thioesterase family protein	_	-121
GJR88_02707	Unknown	hypothetical protein	_	98
GJR88_03054	Unknown	hypothetical protein	SRRM1, SRM160	-51
GJR88_03450	Unknown	hypothetical protein	_	-83
GJR88_03512	Unknown	uncharacterized membrane protein	_	-33
GJR88_04027	Unknown	bile acid 7-alpha dehydratase	_	26
GJR88_04163	Unknown	ferredoxin	_	-95
GJR88_04349	Unknown	alpha/beta hydrolase	*cpo*	24
GJR88_04473	Unknown	nudix superfamily hydrolase	_	320
GJR88_04535	Unknown	hypothetical protein	_	-174
GJR88_04550	Unknown	hypothetical protein	_	147

GJR88_04551	Unknown	membrane protein	MYO15	-323
GJR88_04556	Unknown	phosphotransferase enzyme family protein, protein kinase	_	-22
GJR88_04622	Unknown	hypothetical protein	_	158
GJR88_04623	Unknown	hypothetical protein	_	-50
GJR88_04644	Unknown	putative peroxidase	E1.11.1.19	23
GJR88_04710	Unknown	hypothetical protein	_	180
GJR88_04754	Unknown	hypothetical protein	_	-159
GJR88_04792	Unknown	hypothetical protein	_	123
GJR88_04880	Unknown	N-acetyltransferase GCN5	_	367
GJR88_05044	Unknown	hypothetical protein	_	58
GJR88_05051	Unknown	putative esterase	_	-144
GJR88_05328	Unknown	aldehyde dehydrogenase	E1.2.1.3	73
GJR88_05330	Unknown	acyl-CoA dehydrogenase	_	-42
